# ﻿Integrative review of *Xylomoiastrix*, *X.retinax* and *X.stangelmaieri* (Lepidoptera, Noctuidae, Xyleninae, Apameini)

**DOI:** 10.3897/zookeys.1221.132205

**Published:** 2024-12-23

**Authors:** Risto Haverinen, Aleksander Pototski, Marko Mutanen, Darius Mikalauskas, Roman V. Yakovlev, Günter C. Müller, Alexey M. Prozorov, Aidas Saldaitis

**Affiliations:** 1 Ripako Oy, Vantaa, Finland Ripako Oy Vantaa Finland; 2 Estonian Society of Lepidopterologists, Tallinn, Estonia Estonian Society of Lepidopterologists Tallinn Estonia; 3 Lasnamäe Gymnasium, Tallinn, Estonia Lasnamäe Gymnasium Tallinn Estonia; 4 Ecology and Genetics Research Unit, P.O. Box 3000, FI-90014 University of Oulu, Oulu, Finland University of Oulu Oulu Finland; 5 Lithuanian Entomological Society, Akademijos str. 2, 08412 Vilnius-21, Lithuania Lithuanian Entomological Society Vilnius Lithuania; 6 Laboratory of Biodiversity and Ecology, Tomsk State University, Lenina pr. 36, RUS-634050 Tomsk, Russia Tomsk State University Tomsk Russia; 7 Western Caspian University, Istiglaliyyat Street, 31. Baku 1001, Azerbaijan Western Caspian University Baku Azerbaijan; 8 Samarkand State University, University blv. 15, 140104 Samarkand, Uzbekistan Samarkand State University Samarkand Uzbekistan; 9 University of Sciences, Techniques and Technologies of Bamako, BP 1805 Bamako, Mali University of Sciences Bamako Mali; 10 Kuvin Center for the Study of Infectious and Tropical Diseases, Hadassah Medical School, The Hebrew University, Kalman Ya’akov Man St., 91120 Jerusalem, Israel The Hebrew University Jerusalem Israel; 11 Ludwig-Maximilians-University of Munich, Großhaderner str. 2, D-82152 Planegg-Martinsried, Germany Ludwig-Maximilians-University of Munich Planegg-Martinsried Germany; 12 Bavarian Natural History Collections (SNSB-ZSM), Münchhausen str. 21, D-81247 Munich, Germany Bavarian Natural History Collections Munich Germany; 13 Nature Research Centre, Akademijos str. 2, 08412 Vilnius-21, Lithuania Nature Research Centre Vilnius Lithuania

**Keywords:** DNA barcoding, European fauna, morphology, new status, Palearctic

## Abstract

The relationship of *Xylomoiastrix* Mikkola, 1980; *Xylomoiaretinax* Mikkola, 1998; and *Xylomoiastangelmaieri* Mikkola, 1998 is reconsidered based on 59 genitalia slides (37 males and 22 females) and 40 barcodes of adults collected from the type localities and areas in-between. Due to lack of stable morphologic differences, apart from the wing coloration of *X.retinax*, and low genetic distance between the three, they are considered as three subspecies of *X.strix*: the nominotypical one *X.strixstangelmaieri***stat. nov.** and *X.strixretinax***stat. nov.** Included are photographs of all specimens covering 37 adults, and 28 male and 18 female genitalia, as well as a phylogenetic tree and a map showing collecting localities.

*The article is dedicated to Kari Nupponen (15.01.1962–2.12.2021), a Finnish lepidopterologist, whose main interest was in the family Scythrididae. The first two authors of the article participated in many joint expeditions, traveling together with Kari around the world for nearly twenty years.*

## ﻿Introduction

*Xylomoia* Staudinger, 1892 is a Holarctic genus from the tribe Apameini containing eight species (Mikkola 1998; Lafontaine and Schmidt 2010; Kononenko 2016a, 2016b): 1) *X.chagnoni* Barnes & McDunnough, 1917; type locality (TL): Canada, “Quebec, Rouville Co. and Mt St Hilaire;” 2) *X.indirecta* (Grote, 1875); TL: Canada, “British Columbia, Vancouver Island;” 3) *X.apameaoides* (Hacker, 1989); TL: Turkey, “Prov. Hakkari, Yüksekova;” 4) *X.fusei* Sugi, 1976; TL: Japan, “Gumma Pref., Itakura;” 5) *X.graminea* (Graeser, 1889); TL: “Russia, Amurland, Khabarofka;” 6) *X.strix* Mikkola, 1980; TL: “Latvia, Turaida;” 7) *X.retinax* Mikkola, 1998; TL: “Russia, Western Siberia, Akademgorodok (40 km SE Novosibirsk);” and 8) *X.stangelmaieri* Mikkola, 1998; TL: “N Italy, Venezia Giulia, Caorle.” The latter three, here termed the *strix* group, are evidently very closely related and are of particular interest.

*Xylomoiastrix* is a widespread European species recorded for Finland, Estonia, Latvia, Lithuania, Poland, Belarus, Ukraine, and European Russia (Mikkola 1980; Šulcs and Šulcs 1983; Skou 1991; Nowacki and Sekuła 1994; Karvonen 1996; Klyuchko et al. 2001; Zilli et al. 2005; Savenkov and Šulcs 2010; Pekarsky and Korb 2012 as *X.retinax*; Ivinskis and Rimšaitė 2013; Sachkov 2013; Nowacki and Pałka 2014; Haverinen et al. 2016; Aarvik et al. 2017; Anikin et al. 2017; Geryak et al. 2018; Ūsaitis et al. 2019; Derzhinsky 2019; Matov et al. 2019, 2023; Bolshakov and Makarichev 2020; Haverinen et al. 2021). *Xylomoiaretinax* is recorded from Irkutsk westwards to Novosibirsk, Omsk, Chelyabinsk, Yaroslavl, and is also found in Altai Republic in Russia (Mikkola 1998; Nupponen and Fibiger 2002; Sviridov 2002; Zilli et al. 2005; Knyazev et al. 2015, 2016; Volynkin and Ivanova 2016; Matov et al. 2019, 2023; Knyazev 2020). The border between two species seems to lie between the Volga River and Ural Mountains but it is not precisely defined: specimens originated from Tatarstan, Samara, and Saratov Oblasts were identified as *X.strix* (e.g., Matov et al. 2019, 2023), while specimens collected approximately 500 km eastwards from the Volga River, near Miass in Chelyabinsk Oblast, were attributed to *X.retinax* (Mikkola 1998). *Xylomoiastangelmaieri* is even rarer, it is only known from around the type locality, the Adriatic coast near Venice in northern Italy, and is unknown elsewhere (Mikkola 1998).

**Figures 1–4.**
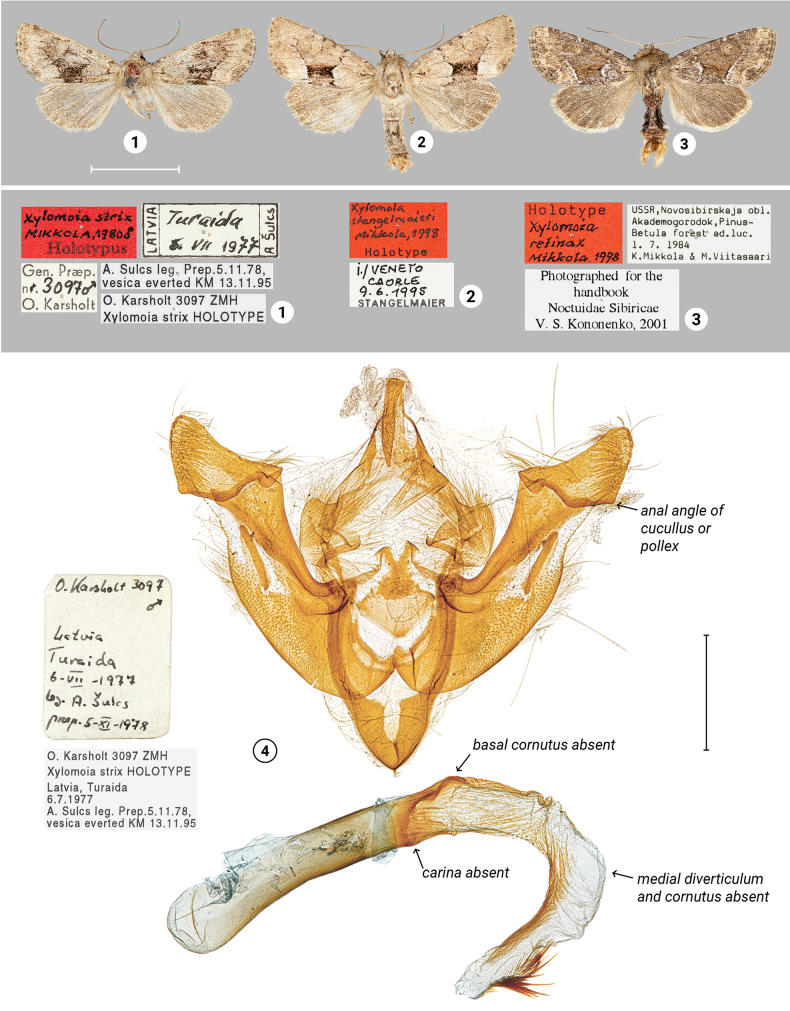
Adults and male genitalia of *Xylomoia* spp. with labels (ZMHF). **1, 4**HT male of *X.strix*, lacking features of phallus are indicated with arrows **2**HT male of *X.stangelmaieri***3**HT male of *X.retinax*. Scale bars: 1 cm (for adults); 1 mm (for genitalia).

The primary types of *X.strix* (Fig. 1) and *X.stangelmaieri* (Fig. 2) are similar in appearance, while *X.retinax* (Fig. 3) is darker than the other two and lacks the dark contrasting pattern in the medial field of the forewing. Among the holotype males, genitalia were studied only for *X.strix*, whereas paratype males were dissected for *X.retinax* and *X.stangelmaieri*. The phallus of the holotype specimen of *X.strix* lacks “the basal cornutus/spines of the vesica, and the medial diverticulum and cornutus of it, present in all other species of the clade” (Mikkola 1998). Later publications do not contain a description of the phallus of any other *X.strix*. Phalli of the paratype males of *X.retinax* and *X.stangelmaieri*, in contrary to *X.strix*, do have the ventral spines of phallus (also called *carina*), and basal and medial cornuti – as in the original description. Instead of morphological investigation, the before-mentioned authors (except Mikkola 1998) focused on the biology and ecology of *X.strix*, leaving unclear whether its holotype exhibits a unique aberration or the species as a whole lacks the spiky features on phallus. Morphological variability of *X.retinax* and *X.stangelmaieri* also was not thoroughly studied. Sviridov (2002), for instance, mentioned that specimens of *X.retinax* from Yaroslavl Oblast have a curved medial cornutus, which he considered a potential reason to establish a new subspecies and suggested that it was in need of detailed investigation. To understand the morphological variability of each taxon, its distribution area, and taxonomic status, a large quantity of adults had to be accumulated. We analyze published data and add original discoveries in morphology, phylogeny, and natural history of the *X.strix* group and reconsider the systematic position of the related taxa.

Abbreviations of the depositories used:

**ASV** collection of A. Saldaitis (Vilnius, Lithuania);

**CEO** collection of E. Õunap (Tartu, Estonia);

**CJK** collection of J. Karvonen (Helsinki, Finland);

**CKTN** collection of K. & T. Nupponen (Espoo, Finland);

**CKP** collection of K. Pałka (Lublin, Poland);

**CMR** collection of M. Rantala (Kerava, Finland);

**CNC**Canadian National Collection of Insects, Arachnids and Nematodes (Montreal, QC, Canada);

**CPI** collection of P. Ivinskis (Vilnius, Lithuania);

**CRH** collection of R. Haverinen (Vantaa, Finland);

**DMV** collection of D. Mikalauskas (Vilnius, Lithuania);

**PFC**Canadian Forest Service, Pacific Forestry Centre (Victoria, BC, Canada);

**ZMHF**Zoological Museum, University of Helsinki (Finland);

**ZMUO**collection of Zoological Museum of the University of Oulu (Finland).

Other abbreviations used:

**GS** genitalia slide;

**HT** holotype;

**PT** paratype;

**TL** type locality.

## ﻿Materials and methods

Adults were photographed with a Nikon D3300, a Nikon 40mm f/2.8G and a Nikon R1C1. Slides were photographed using a Leica MC170 HD. All images were processed with Photoshop CS6, and color plates were made with InDesign CS6.

Genitalia preparations were made following Hardwick (1950). The distal one third of the abdomen of each specimen was put into a separate 50 ml Falcon tube with 10 ml of 13% solution of potassium hydroxide (KOH). Several tubes with abdomens and KOH were placed into a small pot with hot water for 20 min. The tubes thereafter were removed from the pot and the abdomens were rinsed with water several times to remove any remaining scales and soft tissue. Cleaned abdominal parts were then transferred into separate cells of the Corning Costar 96 Well Cell Culture Cluster with a small quantity of water to keep them moist during preparation. Sequentially, abdomens were cleaned with a soft brush and dissected using Dumont Tweezers Style 5 and micro scissors in a Petri dish under the microscope. The phallus was extracted and vesica everted with an insulin syringe and a 32G or 33G needle for mesotherapy. The vesica was stained with Evans blue (Evans and Schulemann 1914; Cooksey 2013). The dissected genitalia were rinsed in 50, 70, and 96% ethanol and then mounted on a microscope slide in Euparal and covered with a cover slip. Morphological terminology partially follows Pierce (1909), Mikkola (1998), and Volynkin (2024).

COI barcodes of 46 specimens from BOLD projects were used for this study (Ratnasingham and Hebert 2007, 2013). The samples were collected in seven countries and stored in nine entomological collections (Table 1). One leg from each individual was used for analysis. Legs were stored in tubes with 96% ethanol. The sequences were obtained at the Biodiversity Institute of Ontario, Canada. DNA isolation, PCR amplification, and DNA sequencing followed standard protocols (Hebert et al. 2003; deWaard et al. 2008).

**Table 1.** Data on specimens and their barcodes deposited in BOLD and used in the phylogenetic analysis.

Taxon / BIN number	#	Process ID / Sample ID	Specimen details and collecting data (depository)
*X.strixstangelmaieri* / BOLD:ABA9763	1	LEFIJ4675-16 / KN00913	male, **Italy**, Veneto, Valle Vecchia, 45.616°N, 12.916°E, 3 m, 15.04.2015, leg. R. Haverinen (CKTN)
	2	LEFIJ4676-16 / KN00914	female, **Italy**, Veneto, Valle Vecchia, 45.616°N, 12.916°E, 3 m, 15.04.2015, leg. R. Haverinen (CKTN)
	3	LEFIJ4677-16 / KN00915	male, **Italy**, Veneto, Valle Vecchia, 45.616°N, 12.916°E, 3 m, 15.04.2015, leg. R. Haverinen (CKTN)
	4	LEFIJ7558-18 / MM24198	female, **Italy**, Veneto, Valle Vecchia, 45.6167°N, 12.9333°E, 3 m, 16.04.2014, leg. R. Haverinen and M. Hirvonen (CRH)
	5	LEFIJ7559-18 / MM24199	male, **Italy**, Veneto, Valle Vecchia, 45.6167°N, 12.9333°E, 3 m, 16.04.2014, leg. R. Haverinen and M. Hirvonen (CRH)
	6	LEFIJ7560-18 / MM24200	male, **Italy**, Veneto, Valle Vecchia, 45.6167°N, 12.9333°E, 3 m, 16.04.2014, leg. R. Haverinen and M. Hirvonen (CRH)
	7	LEPAL476-17 / MM06019	female, **Italy**, Veneto, Valle Vecchia, 45.61°N, 12.93°E, 3 m, 29.06.2014, leg. R. Haverinen and M. Hirvonen (CRH)
	8	LEPAL482-17 / MM24002	male, **Italy**, Veneto, Valle Vecchia, 45.61°N, 12.93°E, 3 m, 15.06.2014, leg. R. Haverinen and M. Hirvonen (CRH)
*X.strixstrix* / BOLD:ADA4423	9	LEFID225-10 / MM06083	male, **Latvia**, Turaida, leg. R. Haverinen (ZMUO)
	10	LEFIJ4666-16 / MM25269	adult, **Finland**, Nylandia, Hanko, 65.0158°N, 25.6574°E, 15.07.1994, leg. J. Karvonen (CJK)
	11	LEFIJ4668-16 / KN00906	male, **Latvia**, Turaida, 57.166°N, 24.85°E, 20 m, 30.06.2005, leg. T. Nupponen (CKTN)
	12	LEFIJ4669-16 / KN00907	female, **Latvia**, Turaida, 57.166°N, 24.85°E, 20 m, 7.07.2005, leg. K. Nupponen (CKTN)
	13	LEFIJ7512-18 / MM24023	adult, **Estonia**, Misso, 58.6481°N, 25.9169°E, 3.07.2012, leg. E. Õunap (CEO)
	14	LEFIJ7513-18 / MM24024	adult, **Estonia**, Misso, 58.6481°N, 25.9169°E, 3.07.2012, leg. E. Õunap (CEO)
	15	LEFIJ7544-18 / MM24106	larva, **Russia**, Lotoshinskyi district, Moscow region, Sevastino village, 56.3877°N, 35.7431°E, 20.08.2014, leg. A. Komrakov (ZMOU)
	16	LEFIJ7561-18 / MM24201	male, **Russia**, Saratov district, settlement Zonalny, 51.5833°N, 46.1°E, 15 m, 16.05.2014, leg. R. Haverinen and A.Belik (CRH)
	17	LEFIJ7562-18 / MM24202	male, **Russia**, Saratov district, settlement Zonalny, 51.5833°N, 46.1°E, 15 m, 16.05.2014, leg. R. Haverinen and A.Belik (CRH)
	18	LEFIJ7563-18 / MM24203	female, **Russia**, Saratov district, settlement Zonalny, 51.5833°N, 46.1°E, 15 m, 16.05.2014, leg. R. Haverinen and A.Belik (CRH)
	19	LEFIJ7564-18 / MM24204	female, **Russia**, Saratov district, settlement Zonalny, 51.5833°N, 46.1°E, 15 m, 16.05.2014, leg. R. Haverinen and A.Belik (CRH)
	20	LEFIJ7565-18 / MM24205	female, **Russia**, Saratov district, settlement Zonalny, 51.5833°N, 46.1°E, 15 m, 16.05.2014, leg. R. Haverinen and A.Belik (CRH)
	21	LEFIJ21338-21 / MM27347	male, **Russia**, Orenburgskaya Oblast, near Kuvandyk village, 225 m, 25.06.2019, leg. M. Rantala (CMR)
	22	LEFIJ21339-21 / MM27348	female, **Russia**, Orenburgskaya Oblast, near Kuvandyk village, 225 m, 25.06.2019, leg. M. Rantala (CMR)
	23	LEPAL477-17 / MM06020	male, **Poland**, Skvyhiozyn, 52.0685°N, 19.4357°E, 16.04.2014, leg. K. Pałka (CKP)
	24	LEPAL478-17 / MM06021	male, **Poland**, Skvyhiozyn, 52.0685°N, 19.4357°E, 20.05.2013, leg. K. Pałka (CKP)
	25	LEPAL479-17 / MM06022	male, **Poland**, Malice, 52.0685°N, 19.4357°E, 23.05.2014, leg. K. Pałka (CKP)
	26	LEPAL480-17 / MM06023	male, **Poland**, Malice, 52.0685°N, 19.4357°E, 16.05.2014, leg. K. Pałka (CKP)
	27	LEPAL481-17 / MM24001	male, **Estonia**, vs Valga, Koiva River, Koikküla, 57.63 N, 26.23 E, 16.05.2014, leg. R. Haverinen (CRH)
*X.strixstrix* / BOLD:ADA4423	28	LEPAL483-17 / MM24003	female, **Estonia**, Põlvamaa, Veski, 57.83°N, 27.51°E, 15.06.2014, leg. R. Haverinen (CRH)
	29	LEPAL484-17 / MM24004	male, **Estonia**, Põlvamaa, Veski, 57.83°N, 27.51°E, 16.04.2014, leg. R. Haverinen (CRH)
	30	LEPAL485-17 / MM24005	female, **Russia**, Saratov district, settlement Zonalny, 51.58 N, 46.1 E, 20.06.2014, leg. R. Haverinen, K. Nupponen, A. Pototski and A. Belik (CRH)
	31	LEPAL486-17 / MM24006	male, **Russia**, Saratov district, settlement Zonalny, 51.58°N, 46.1°E, 20.06.2014, leg. R. Haverinen, K. Nupponen, A. Pototski and A. Belik (CRH)
	32	LEPAL487-17 / MM24007	male, **Estonia**, Saaremaa, Kogula, 58.28°N, 22.25°E, 19.06.2014, leg. R. Haverinen (CRH)
	33	LEPAL488-17 / MM24008	male, **Estonia**, Saaremaa, Kogula, 58.28°N, 22.25°E, 19.06.2014, leg. R. Haverinen (CRH)
	34	LEPAL489-17 / MM24021	larva, **Estonia**, Koiva River, Koikküla, 58.6481°N, 25.9169°E, 24.08.2014, leg. R. Haverinen (CRH)
*X.strixretinax* / BOLD:ADA4423	35	LEFIJ4670-16 / KN00908	male, **Russia**, Novosibirsk district, Novosibirsk, Akademgorodok, 59.0394°N, 98.6705°E, 110 m, 13.09.2014, leg. R. Haverinen and A. Pototski (CKTN)
	36	LEFIJ4671-16 / KN00909	male, **Russia**, Novosibirsk district, Novosibirsk, Akademgorodok, 59.0394°N, 98.6705°E, 110 m, 13.09.2014, leg. R. Haverinen and A. Pototski (CKTN)
	37	LEFIJ4672-16 / KN00910	female, **Russia**, Novosibirsk district, Novosibirsk, Akademgorodok, 59.0394°N, 98.6705°E, 110 m, 13.09.2014, leg. R. Haverinen and A. Pototski (CKTN)
	38	LEFIJ4673-16 / KN00911	male, **Russia**, Novosibirsk district, Novosibirsk, Akademgorodok, 59.0394°N, 98.6705°E, 110 m, 13.09.2014, leg. R. Haverinen and A. Pototski (CKTN)
	39	LEFIJ4674-16 / KN00912	female, **Russia**, Novosibirsk district, Novosibirsk, Akademgorodok, 59.0394°N, 98.6705°E, 110 m, 13.09.2014, leg. R. Haverinen and A. Pototski (CKTN)
	40	LEFIJ7511-18 / MM24022	larva, **Russia**, Novosibirsk, 59.0394°N, 98.6705°E, leg. R. Haverinen and A. Pototski (CRH)
*X.graminea* / BOLD:ADN5882	41	LEFIJ7545-18 / MM24107	male, **Lithuania**, Kalniskes, 55.2944°N, 23.946°E, 21.06.2013, leg. P. Ivinskis (ZMUO)
	42	LEFIJ7546-18 / MM24108	**Lithuania**, Kalniskes, 55.2944°N, 23.946°E, 21.06.2013, leg. P. Ivinskis
*X.chagnoni* / BOLD:AAE4227	43	RDNMG580-08 / CNC LEP00052404	adult, **Canada**, Ontario, Stittsville, 45.2005°N, 75.98°W, 131.066 m, 4.07.2003, leg. J. Troubridge (CNC)
	44	RDNMG581-08 / CNC LEP00052405	adult, **Canada**, Ontario, Stittsville, 45.2005°N, 75.98°W, 131.066 m, 15.07.2003, leg. J. Troubridge (CNC)
*X.indirecta* / BOLD:AAB1776	45	LHLEP387-06 / UBC-2006-1537	male, **Canada**, British Columbia, Maple Ridge, UBC Research Forest, 49.266°N, 122.573°W, 158 m, 1.08.2006, leg. A. Li and J. Derhousoff (PFC)
	46	LHLEP388-06 / UBC-2006-1538	male, **Canada**, British Columbia, Maple Ridge, UBC Research Forest, 49.266°N, 122.573°W, 158 m, 1.08.2006, leg. A. Li and J. Derhousoff (PFC)

Sequence alignment and calculation of pairwise distances were conducted using MEGA X (Kumar et al. 2018). Maximum Likelihood (ML) analysis of the aligned COI sequences was conducted using IQ-TREE 2.2.0 (Minh et al. 2020) under HKY+F+I nucleotide substitution model as preferred to by ModelFinder (Kalyaanamoorthy et al. 2017), and with 1000 ultrafast bootstrap replicates. The tree rooted to *X.chagnoni* + *X.indirecta* was constructed using FigTree 1.4.4 and polished with CorelDraw 24.5.0.731 and InDesign CC 2019.

Map of ecoregions was taken from ecoregions.appspot.com (see Dinerstein et al. 2017).

### ﻿Review of morphology

***Wing coloration*** (Figs 1–3, 5–41). In general, two types of wing coloration are distinguished: 1) *X.stangelmaieri* + *X.strix* with a dark area in the medial field, and 2) *X.retinax* without a dark area in the medial field. *Xylomoiastangelmaieri* has a narrow blackish streak with reddish brown margins (Figs 5–10), whereas *X.strix* has this streak varying from narrow to wide with more or less pronounced reddish-brown edges. It may expand towards the costa covering medial field (Figs 11–15, 19, 26). Otherwise, all three species are similar. Tinge of wing coloration does vary from greyish to brownish even in adults collected from the same location. This may be due to some variety of mineral composition of the soil that is picked up by the host plant.

***Male genitalia*** (Figs 42–68). Four of the five genitalia of *X.stangelmaieri* exhibit an anal angle of cucullus (*pollex* sensu Pierce 1909; also see Volynkin 2024) which is better pronounced than in *X.strix* and *X.retinax* (also see Mikkola 1998). In addition, the shape of the uncus, valva, and saccus as well as the presence or absence of a carina, the basal or medial cornuti on the phallus vary within each species. Such variability is not exclusive for *Xylomoia* and is known for other Apameini like *Hydraecia* Guenée, 1841 and *Photedes* Lederer, 1857.

***Female genitalia*** (Figs 69–89). *Xylomoiaretinax* lacks fold of ductus bursae (Figs 87–89), whereas both *X.stangelmaieri* and *X.strix* exhibit it. Otherwise, shape of the bursa copulatrix and number of signa vary within each species.

*Xylomoiaretinax* is distinguished from *X.stangelmaieri* and *X.strix* by lack of dark medial field on forewing and lack of fold of ductus bursae in female genitalia; *X.stangelmaieri* is distinguished from *X.strix* and *X.retinax* by a better pronounced pollex on the cucullus (four studied genitalia out of five).

### ﻿Review of phylogeny

The Maximum Likelihood (ML) tree revealed five well-defined clusters (Fig. 90): 1) *X.stangelmaieri*, 2) *X.strix + X.retinax*, 3) *X.graminea*, 4) *X.chagnoni*, and 5) *X.indirecta*. Each of them has high bootstrap value above 80. Both clusters of *X.stangelmaieri* and *X.strix* + *X.retinax* have several weakly supported subclusters that are also unsupported by morphological features or distribution.

Pairwise divergences calculated between *X.stangelmaieri* and *X.strix* vary from 1.48 to 2.3% and between *X.stangelmaieri* and *X.retinax* from 1.37 to 2.13%, whereas *X.strix* and *X.retinax* have a maximum divergence of 0.33% which is reflected in their intermixed positions on the ML tree. All three taxa have 2.13–2.63% *p*-distance from their sister species *X.graminea*. Much higher *p*-distances are calculated between the Eurasian and North American species being running as high as 6.99–8.36%, and *p*-distance between West Canadian *X.indirecta* and East Canadian *X.chagnoni* from 5.93 to 6.23%.

*Xylomoiastrix* and *X.retinax* form a monophyletic clade with a maximum divergence of 0.33% within the clade, whereas *X.stangelmaieri* has an average *p*-distance of 1.84% from the clade *X.strix* + *X.retinax*; *X.graminea* is a sister species to the *X.strix* group with an average *p*-distance of 2.38%; two Canadian species have an average *p*-distance of 7.68% from European taxa and 6.08% between themselves, which is up to 2.5 times higher than between any European taxa.

### ﻿Review of natural history

*Xylomoiastrix* with the closely related *X.retinax* and *X.stangelmaieri* were rather recently described and had remained enigmatic species with unknown biology. The species were only associated with wet habitats near various bodies of water without a particular host plant (Mikkola 1998). Comprehensive investigation of the biology of *X.strix* and its relatives was initiated after 2004, when RH and his daughter Inna found a connection between *X.strix* and *Equisetumhyemale*, apparently the host plant, which was later confirmed by Ahola and Silvonen (2007). Knowing that, RH and AP, together with the late Finnish lepidopterist K. Nupponen, systematically travelled across Europe and to places in Russia for nearly twenty years to unveil the biology and distribution of *X.strix* and its congeners.

Known environments inhabited by *X.strix* in Latvia, Poland, and Ukraine were wetlands, whereas in Estonia the environments were a dry forest meadow and a pine forest (Mikkola 1980; Karvonen 1996). Adults were collected in “deep, dark, wet forest areas close to rivers and or lakes [...] in late June to mid-July” (Zilli et al. 2005). The natural history and distribution of *X.strix* were thoroughly studied in Estonia, where more than 110 localities with growing *E.hyemale* were discovered (Haverinen et al. 2016). More than 80 of them were investigated by RH and AP together with K. Nupponen, and in half of them, *X.strix* was collected. Four field trips were taken to Russia in: 1) 2014 and 2) the first half of May 2015 to Saratov, where caterpillars were found on narrow stems of *E.hyemale* near a growth of *E.hyemale* where stems seemed to be too thin for caterpillars; 3) the first half of May 2015 to Moscow Oblast, where some stems of *E.hyemale* were found with holes bored by caterpillars of *X.strix*; and 4) September 2019 to Luzhsky District of Leningrad Oblast, where two populations of *E.hyemale* were found and a total of 24 caterpillars were collected, from which 16 adults later emerged (Haverinen et al. 2021). The complete life cycle of *X.strix* was described in detail by Haverinen et al. (2016).

Adults of *X.retinax* were collected in: “birch-pine forest at the verge of a slope down to a nearby creek valley” (Mikkola 1998); “old forest patch nearby moist meadow [...] in the end of June – beginning of July” (Zilli et al. 2005); “patch of mixed-grass meadow among ravines abundantly overgrown with sea buckthorn”, and “forest in front of a vast clearing with meadow vegetation” (Knyazev et al. 2015). In mid-September 2014, RH and AP travelled to Novosibirsk, Russia from where only seven specimens of *X.retinax* were known. More than one thousand caterpillars, each inside of an individual stem of long thick plants of *E.hyemale*, were found near Novosibirsk Reservoir and transported to Finland for breeding. Feeding of *X.strix* on *E.hyemale* had also been reported by Knyazev et al. (2016) and Geryak et al. (2018).

The natural history of *X.stangelmaieri* was only known from the original description. Mikkola (1998) wrote that the species was “Found in a wetland habitat on the Adriatic Coast in late May and early June. The moths were caught by light beyond the sandy coastline near marshy lagoons at sea level. The plants in this area included the following (G. Stangelmaier, personal comm.): *Pinuspinea*, *Eleagnusangustifolia*, *Tamarix* sp., *Rubusfruticosus*, *Aristolochia*, *Filipendula*, *Salsola*, *Suaeda*, *Arthrocnemum*, *Crithmum*, *Datura*, *Phragmitescommunis*, *Juncus*, *Typhalatifolia* and *Scirpus*.” The type locality, Valle Vecchia near Venice, Italy, remains the only known locality where *X.stangelmaieri* occurs. RH visited it for the first time in 2007 and subsequently in 2009, when knowledge about the host plant of *X.strix* was shared with G. Stangelmaier and some plants of *Equisetum* damaged by *X.stangelmaieri* were found. Wine-baited traps were set near the type locality in the last week of April 2010 by RH and his daughter, and 49 specimens of *X.stangelmaieri* were collected by J.-P. Kaitila two weeks later. In March–April 2014, RH together with M. Hirvonen found a large number of caterpillars in stems of *Equisetum* plants near Venice: most of them had been collected in a pine forest, while some had been found on dry sand dunes. In the first half of December 2014, RH and AP collected numerous plants with caterpillars and handed them to K. Silvonen and T. Nupponen for breeding. The area was visited again by RH in 2015 and K. Nupponen in 2016. At the end of March 2024 RH, AP, and I. Jürjendal went again to collect *Equisetum* plants to identify the species to which they belonged. They grow up to 150 cm long, may branch, and have thin stems so that caterpillar stretch up to 3–4 cm to fit into them. Plants appeared to be neither *Equisetumramosissimus* nor *E.hyemale*, but, probably, a hybrid or even triploid. Caterpillars of *X.stangelmaieri* deliberately chose *E.hyemale* over another species when offered them in laboratory conditions. They hibernated from mid-November to mid-March in the middle part of the stem in contrary to *X.strix* that overwintered in the lower part of the stems under snow cover. In nature *X.stangelmaeri* may often be parasitized by *Necremnus* sp. (Eulophidae; V. Vikberg, pers. comm. 02 Mar 2015) or eaten by birds, spiders, or black ants (*Myrmica* sp.).

The natural history of other *Xylomoia* species remains relatively unknown, but even these crumbs of information are very important. Bury and Czudec (2019) reared *X.graminea*, a sister species to the *X.strix* group, on *Phragmitesaustralis* under laboratory conditions. They noted that “Just like its related species *X.graminea* is associated with primeval moist habitats, predominantly lush sedge meadows, transitory bogs and rush communities (Buszko 2004, 2010; Bury and Zajda 2012).” Rockburne and Lafontaine (1976) stated that *X.chagnoni*’s host plant was *Phalarisarundinacea*. Both *Phragmitesaustralis* and *Phalarisarundinacea* belong to the family Poaceae, while *E.hyemale*, the host plant of both *X.strix* and *X.retinax*, belongs to the family Equisetaceae. The only cohesive feature of Equisetaceae and Poaceae, in this case, is a meaty stem with an external hard covering suitable for caterpillars to bore through, feed, and develop inside, including safe overwintering. Otherwise, the two families are phylogenetically distant and may be a good differentiating feature to distinguish the groups of species within *Xylomoia*.

*Xylomoiastrix* is included in annexes II and IV of the Council of Europe Directive 92/43/EEC of 21 May 1992 among animal species of Community importance, the preservation of which requires the designation of special protection areas and requires strict protection. In addition, *X.strix* is marked with an asterisk, which means that the species is of a primary importance among the species whose preservation requires the creation of special protected areas (Annex II) and belongs to the list of species in need of strict protection (Annex IV) (Council Directive 1992).

## ﻿Results

Considering similarity of wing coloration (*X.retinax* is distinguished by the lack of a dark medial field), male genitalia (*X.stangelmaieri* is distinguished by bigger pollex), female genitalia (*X.retinax* is distinguished by the lack of fold on ductus bursae), genetic divergence (*X.stangelmaieri* does cluster separately from *X.strix + X.retinax*), and natural history (two of the three species feed on one species of Equisetaceae instead of Poaceae like *X.graminea* and *X.chagnoni*), we suggest all three taxa of the *X.strix* group as populations that still may be undergoing speciation. Two previously established species are downgraded to subspecific status: *X.strixstangelmaieri* stat. nov. and *X.strixretinax* stat. nov. Diagnosis, intrasubspecific variability, and updated distributions are provided below for each subspecies, except for *X.strixstangelmaieri* due to lack of any new collection data.

### ﻿ Xylomoia strix strix

Taxon classificationAnimaliaLepidopteraNoctuidae

Mikkola, 1980

B5409904-0B6C-5FDD-82E2-9B9734284CFA

Figs 1 , 4 , 11–16 , 17–29 , 30–35 , 46–49 , 50–53 , 54–57 , 58–61 , 62–64 , 72–74 , 75–80 , 81–83 , 84–86

Xylomoia strix strix Mikkola, 1980: Notulae Entomologicae 60: 220. TL: “Latvia, Turaida.” Holotype male, ZMHF [examined].

#### Diagnosis.

Distinguished from *X.strixstangelmaieri* by broader dark field on forewings (Figs 11–35) and smaller pollex (Figs 46–64), from *X.strixretinax* by actual presence of dark field on forewing (Figs 11–35) and fold of ductus bursae (Figs 72–86); from both subspecies genetically, having an average *p*-distance of 1.89% from *X.strixstangelmaieri* and 0.33% from *X.strixretinax*. Average *p*-distance between *X.strixstrix* and *X.graminea* is 2.55%, *X.strixstrix* and *X.chagnoni*, 7.64%, and *X.strixstrix* and *X.indirecta*, 8.05% (Fig. 90). Found in north, central, and east Europe with the westernmost presence in the Volga region (Figs 91, 92).

#### Variability.

***Adults.*** Blackish streak in medial field varies from narrow (e.g., Figs 20, 22, 33) to wide (e.g., 18, 23), its reddish-brown bounds vary from well-pronounced (e.g., Figs 15, 21) to non-existing (e.g., Figs 12, 19). Dark streak may expand towards costa and cover medial field (Figs 11–15, 19, 26). Forewings may have somewhat reddish (Figs 16, 23, 30, 33), yellowish (Figs 11–15, 18, 24, 26) or greyish tinge (Figs 22, 25, 27–29, 31–32, 34–35); submarginal field may be pale- (e.g., Fig. 11) or dark-colored (e.g., Fig. 17). ***Male genitalia.*** Uncus may gradually get thin towards apex (e.g., Figs 46, 59) or only be thin near its apex (e.g., Figs 51, 58), saccus may be relatively small and narrow (e.g., Figs 48, 62) or large (e.g., Figs 54, 63), carina may be reduced (Figs 56–57, 59, 61) or well-developed (e.g., Figs 48, 60, 64), basal cornutus varies in size from small (e.g., Fig. 61) to large (e.g., Fig. 58) and may be more or less bent, medial cornutus may be almost straight (e.g., Fig. 46), c-shaped (e.g., Fig. 62) or s-shaped (e.g., Fig. 60) and varies in size. ***Female genitalia.*** Antevaginal plate slightly varies in thickness, bursa copulatrix may narrow around connection with ductus bursae (e.g., Fig. 76) and may have one (Figs 73, 74, 77, 85) or two (Fig. 86) frontal signa, hind signum varies in size.

**Figures 5–16.**
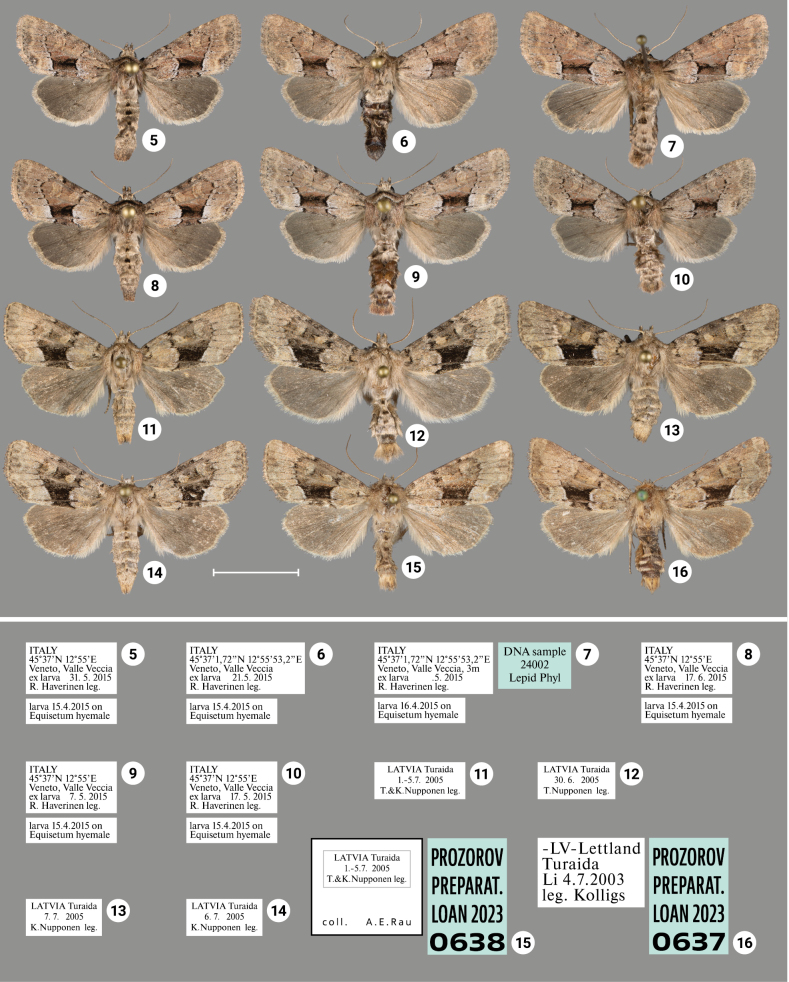
Adults of *Xylomoiastrix* sspp. with labels. **5–10***X.strixstangelmaieri* (CRH) **11–16***X.strixstrix***11–14**CRH**15, 16.**ASV. Scale bar: 1 cm.

**Figures 17–29.**
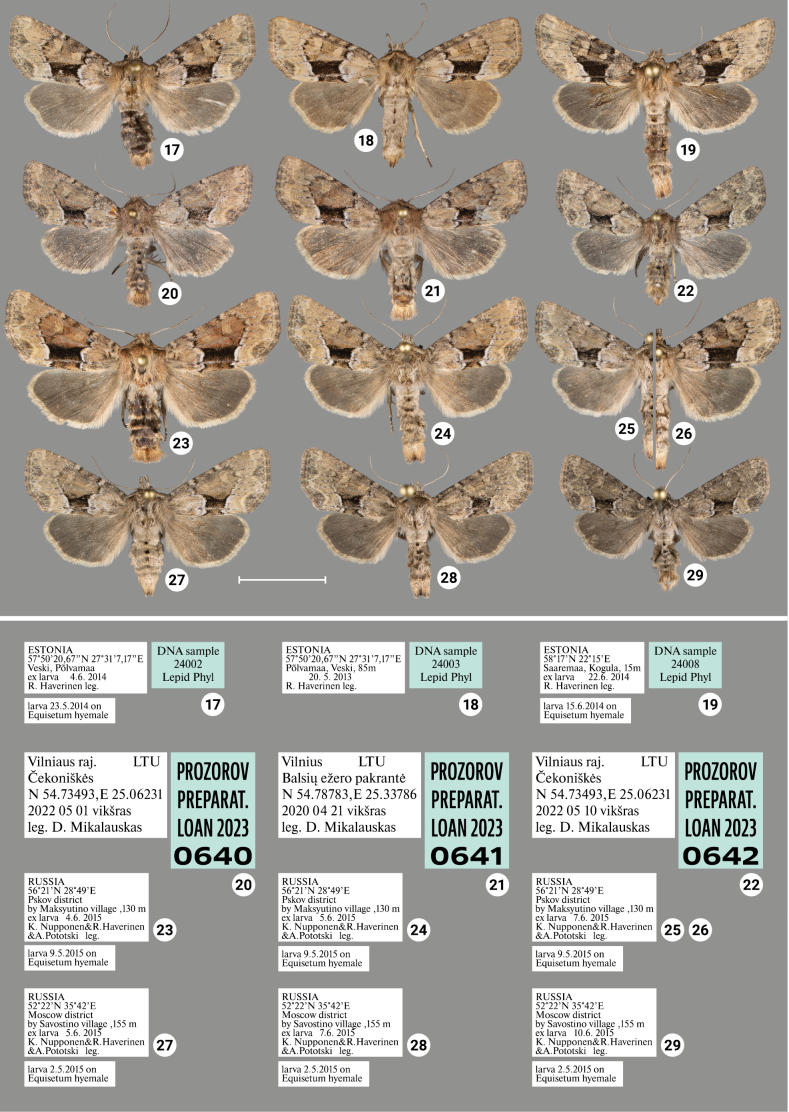
Adults of *Xylomoiastrixstrix* with labels. **17–19, 23–29**CRH**20–22**ASV. Scale bar: 1 cm.

**Figures 30–41.**
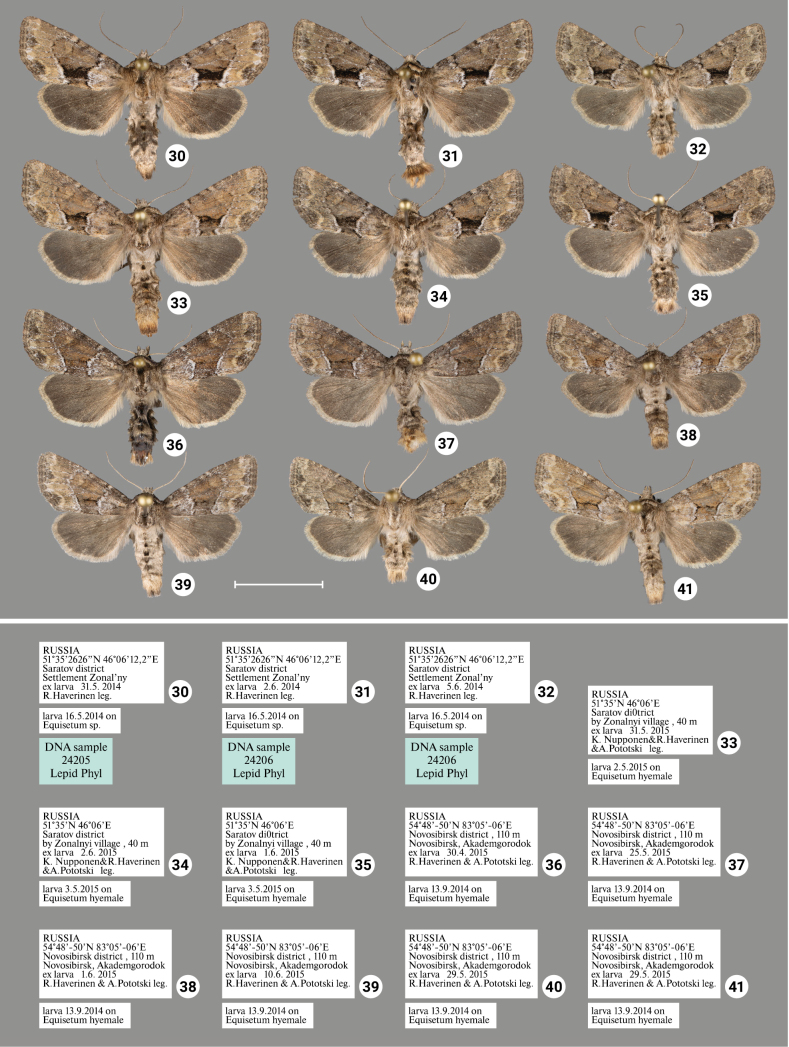
Adults of *Xylomoiastrix* sspp. with labels (CRH). **30–35***X.strixstrix***36–41***X.strixretinax*. Scale bar: 1 cm.

#### Distribution area.

Finland, Estonia, Latvia, Lithuania, Poland, Belarus, Ukraine, and Russia (Leningrad, Yaroslavl, Moscow, Tula, Saratov, Samara Oblasts and Republic of Tatarstan).

### ﻿ Xylomoia strix stangelmaieri

Taxon classificationAnimaliaLepidopteraNoctuidae

Mikkola, 1998 stat. nov.

05CCA8E1-3623-596D-9336-5FE4F237A79B

Figs 2 , 5–10 , 42–45 , 69–71

Xylomoia strix stangelmaieri Mikkola, 1998: Systematic Entomology 23: 182. TL: “N Italy, Venezia Giulia, Caorle.” Holotype male, ZMHF [examined].

#### Diagnosis.

**Figures 42–45.**
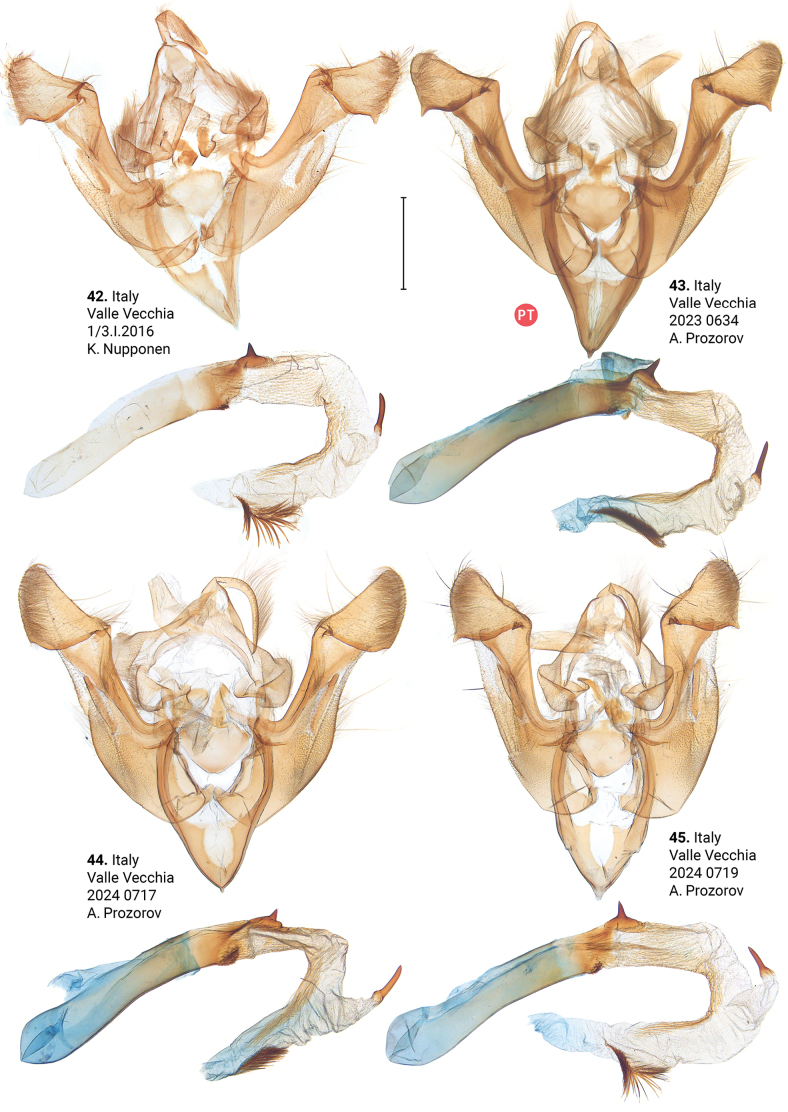
Male genitalia of *Xylomoiastrixstangelmaieri*. Depositories: **42, 44–45**CRH**43**ASV. Scale bar: 1 mm.

Distinguished from *X.strixstrix* by somewhat narrower dark field on forewings and from *X.strixretinax* by actual presence of this dark field (Figs 5–10) and fold of ductus bursae (Figs 69–71); from both subspecies by bigger pollex in male genitalia (Figs 42, 43, 45) and genetically, having an average *p*-distance of 1.89% from *X.strixstrix* and 1.75% from *X.strixretinax*. Average *p*-distance between *X.strixstangelmaieri* and *X.graminea* is 2.28%, *X.strixstangelmaieri* and *X.chagnoni*, 6.77%, *X.strixstangelmaieri* and *X.indirecta*, 8.06% (Fig. 90). Very local, so far found only on the Adriatic coast near Venice in northern Italy (Figs 91, 92).

#### Variability.

***Adults.*** Forewings may have reddish (Figs 5–8) or greyish tinge (Fig. 10), submarginal field may be paler (Figs 6, 7, 9, 10) or darker in color (Figs 5, 8). ***Male genitalia.*** Uncus may gradually narrow towards apex (Fig. 44) or be narrow only near its apex (Figs 42, 43, 45), pollex may be barely noticeable (Fig. 44) or well pronounced (Figs 42, 43, 45), saccus may be narrow (Figs 42, 43) or wide (Figs 44, 45), carina vary in size from small (Fig. 42) to large (Fig. 44), basal cornutus vary in size from small (Fig. 44) to large (Fig. 43), medial cornutus may be straight (Fig. 43) or curved (Figs 42, 44, 45). ***Female genitalia.*** Antevaginal plate may be narrow (Fig. 71) or thick (Fig. 70), bursa copulatrix may be narrow around connection with ductus bursae (Fig. 71); bursa copulatrix may have one (Fig. 70), two (Fig. 69), or three (Fig. 71) frontal signa; hind signum slightly varies in size.

### ﻿ Xylomoia strix retinax

Taxon classificationAnimaliaLepidopteraNoctuidae

Mikkola, 1998 stat. nov.

8AFA9217-4EEB-57FB-B1CC-544B7FBB11A8

Figs 3 , 36–41 , 65–68 , 87–89

Xylomoia strix retinax Mikkola, 1998: Systematic Entomology 23: 181. TL: “Russia, Western Siberia, Akademgorodok (40 km SE Novosibirsk).” Holotype male, ZMHF [examined].

#### Diagnosis.

Distinguished from *X.strixstangelmaieri* by smaller pollex (Figs 65–68), from both congeners by lack of dark medial field on forewing (Figs 36–41), fold of ductus bursae (Figs 87–89) and genetically, having an average *p*-distance of 1.75% from *X.strixstangelmaieri* and 0.33% from *X.strixstrix*. Average *p*-distance between *X.strixretinax* and *X.graminea* is 2.36%, *X.strixretinax* and *X.chagnoni*, 7.22%, and *X.strixretinax* and *X.indirecta*, 8.13% (Fig. 90).

**Figures 46–49.**
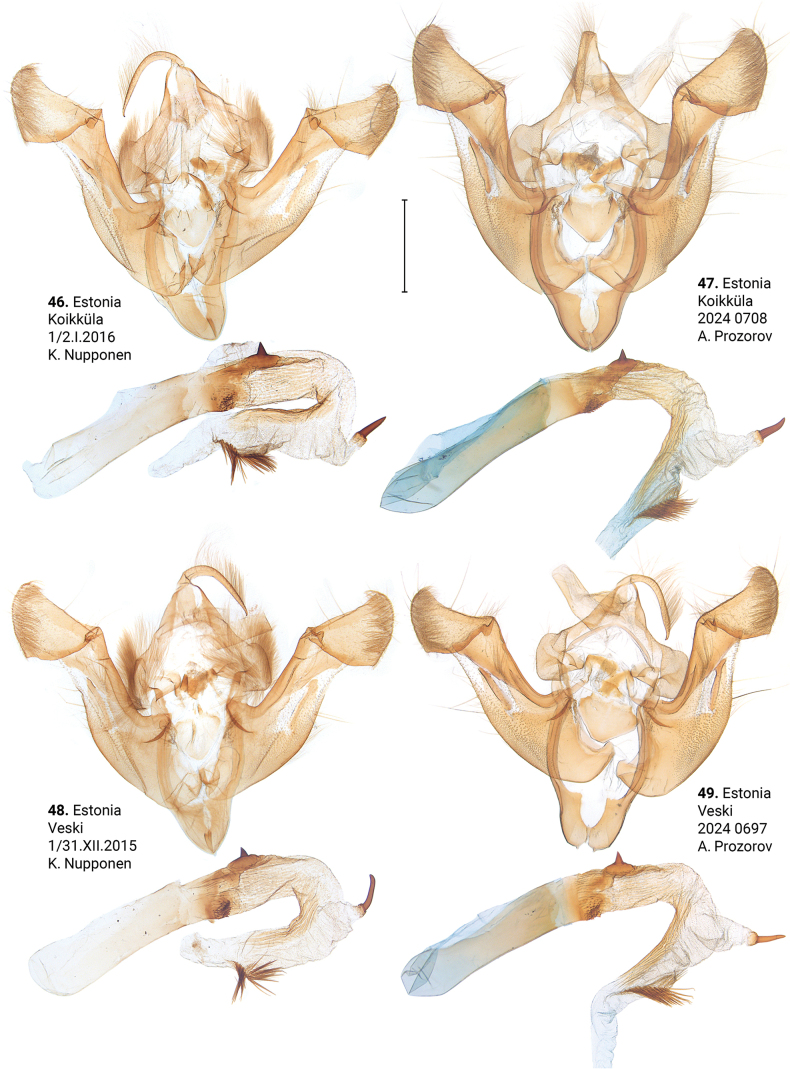
Male genitalia of *Xylomoiastrixstrix* (CRH). Scale bar: 1 mm.

**Figures 50–53.**
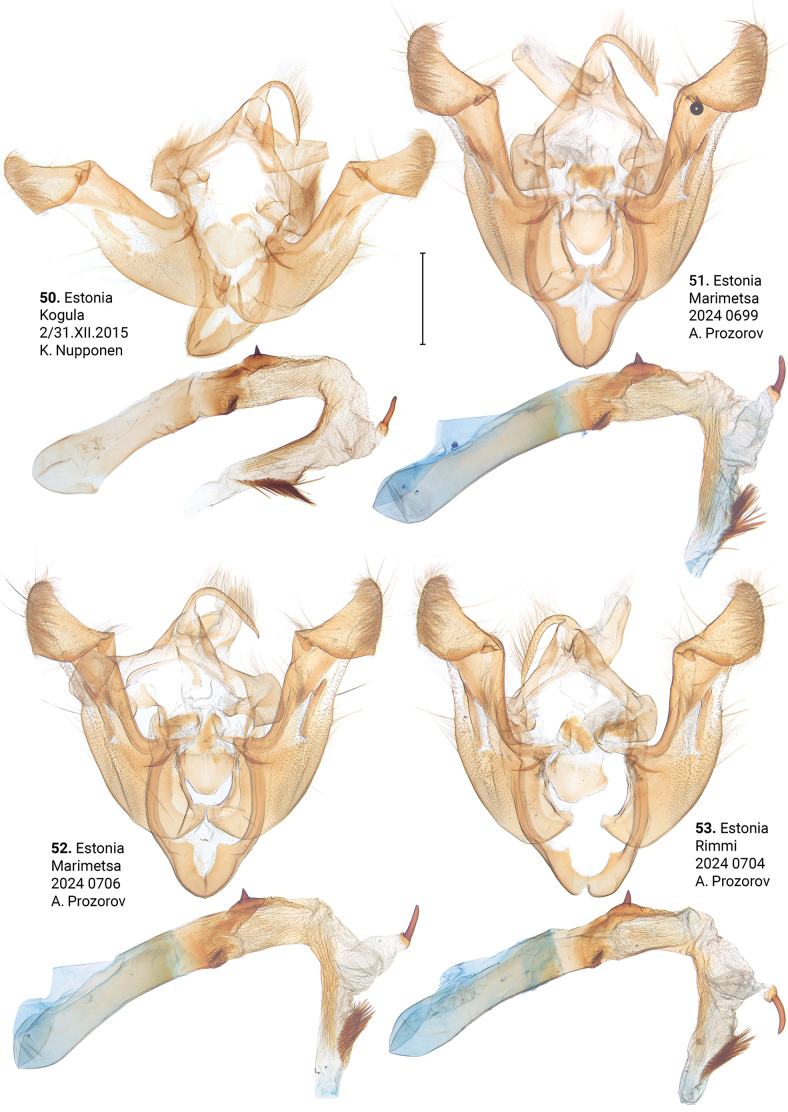
Male genitalia of *Xylomoiastrixstrix* (CRH). Scale bar: 1 mm.

**Figures 54–57.**
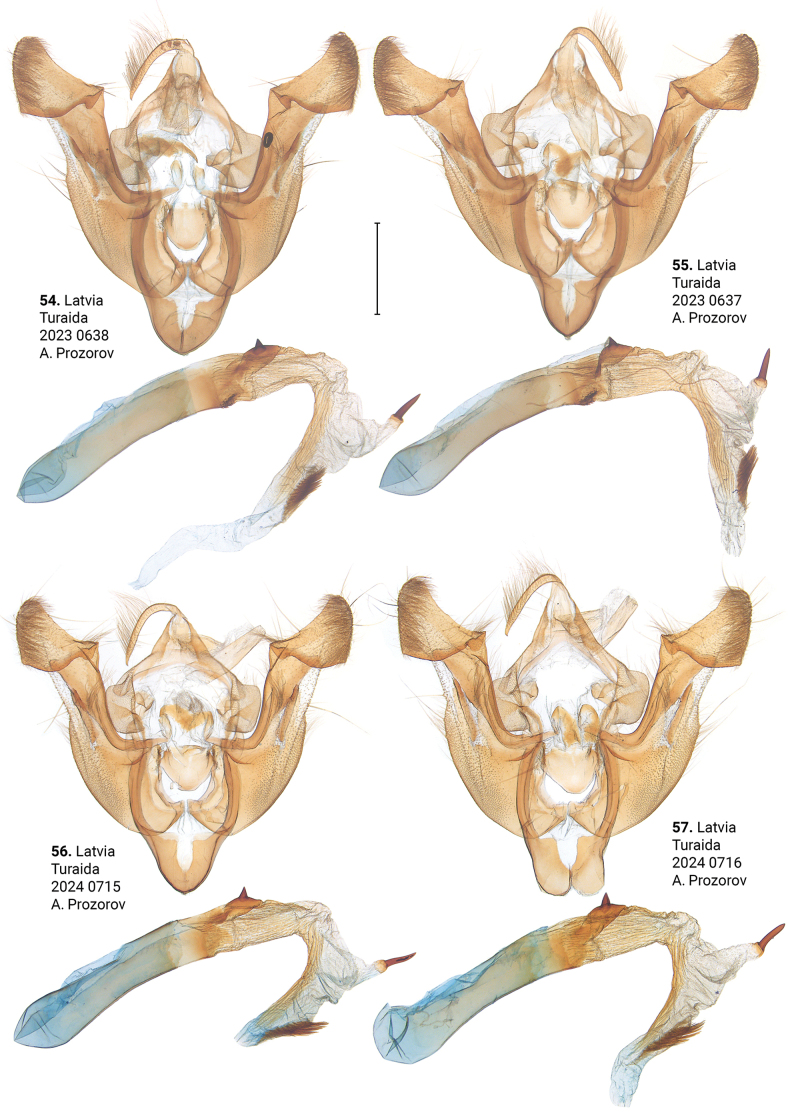
Male genitalia of *Xylomoiastrixstrix*. Depositories: **54, 55**ASV**56, 57**CRH. Scale bar: 1 mm.

**Figures 58–61.**
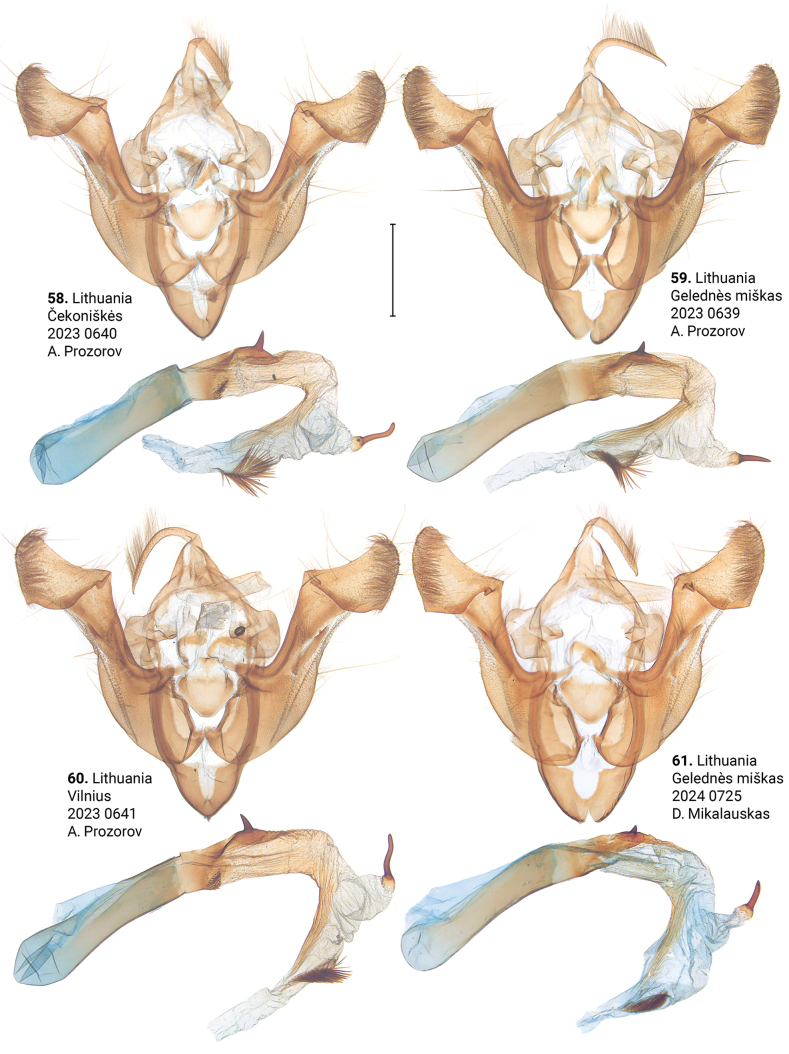
Male genitalia of *Xylomoiastrixstrix* (ASV). Scale bar: 1 mm.

**Figures 62–64.**
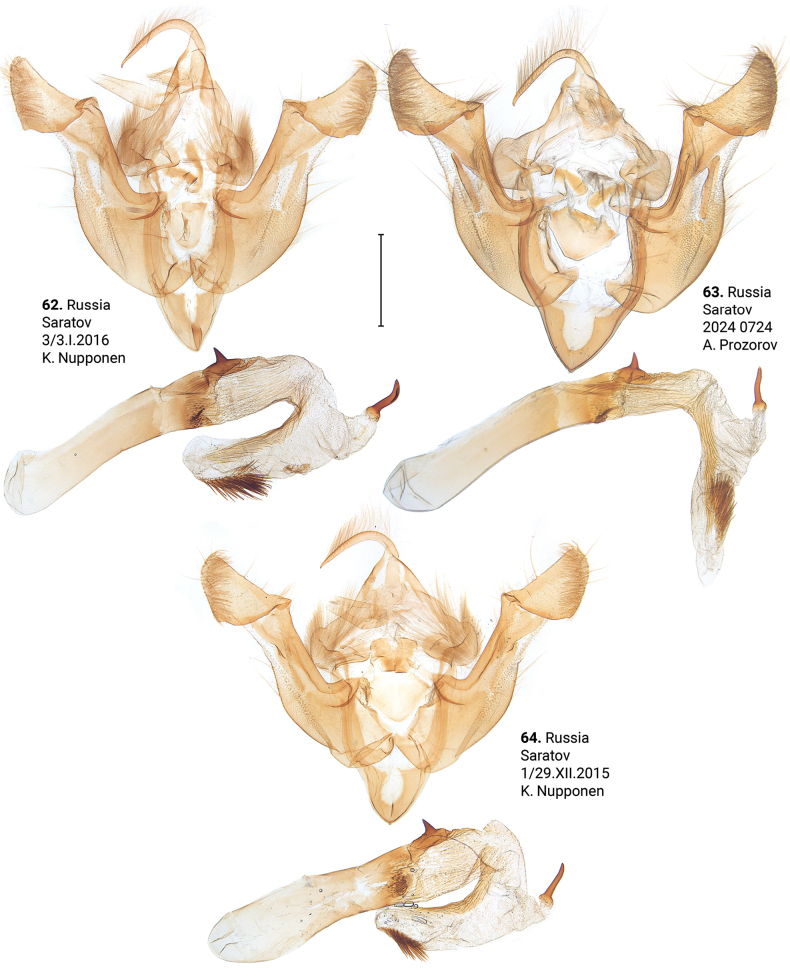
Male genitalia of *Xylomoiastrixstrix* (CRH). Scale bar: 1 mm.

**Figures 65–68.**
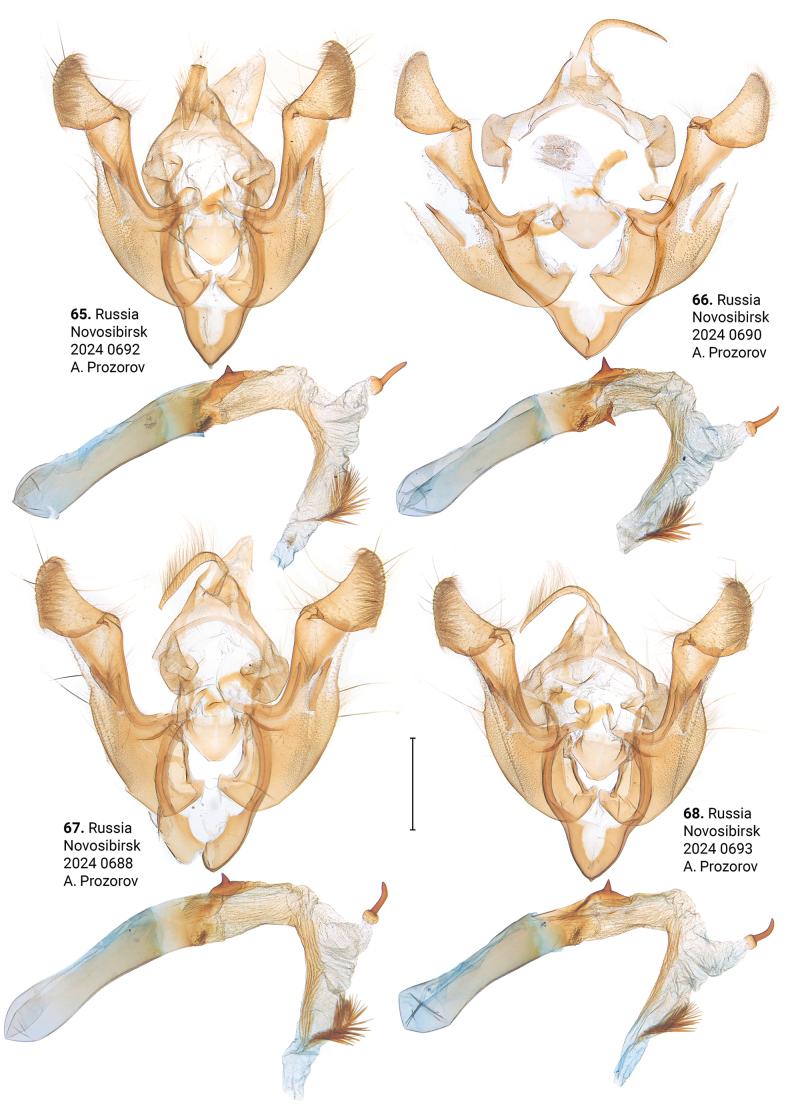
Male genitalia of *Xylomoiastrixretinax* (CRH). Scale bar: 1 mm.

**Figures 69–71.**
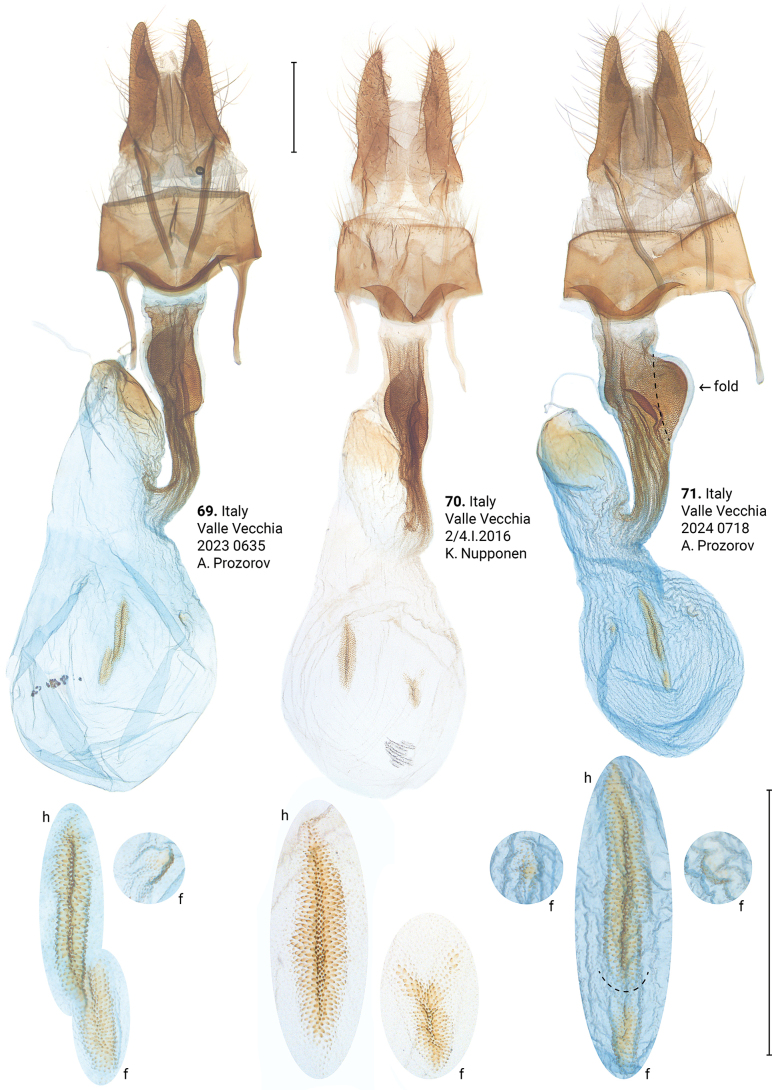
Female genitalia of *Xylomoiastrixstangelmaieri*. Abbreviations: f – frontal signum, h – hind signum. Depositories: **69**ASV**70, 71**CRH. Scale bar: 1 mm.

**Figures 72–74.**
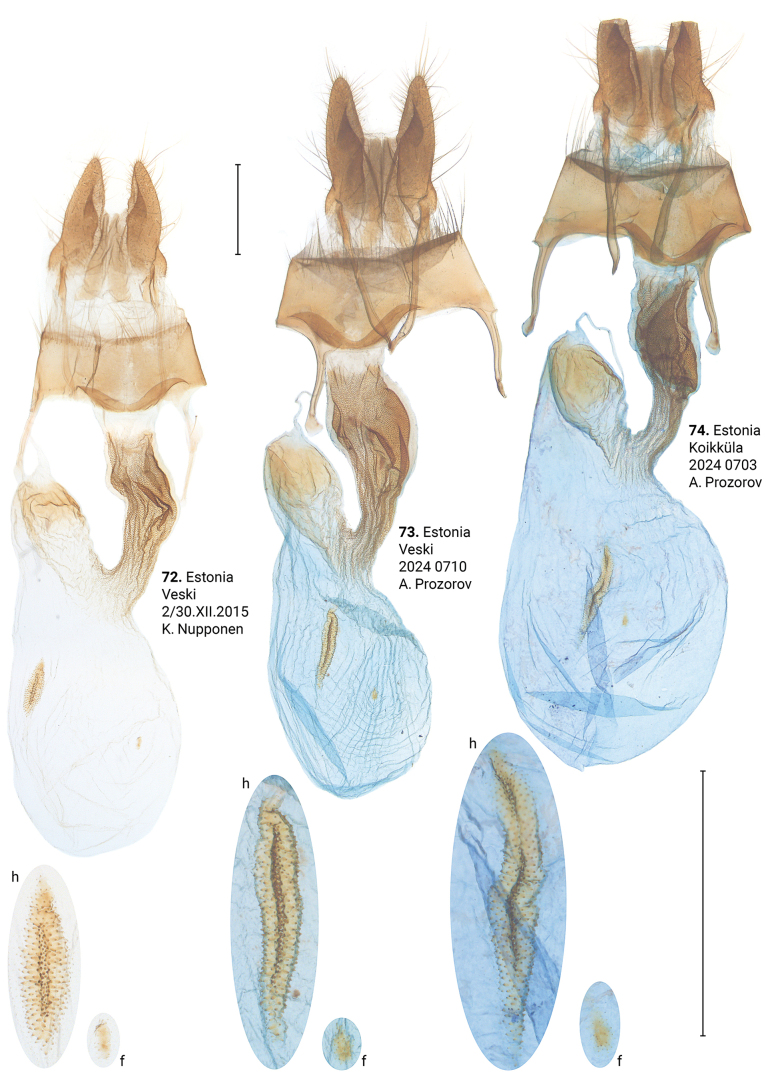
Female genitalia of *Xylomoiastrixstrix* (CRH). Abbreviations: f – frontal signum, h – hind signum. Scale bar: 1 mm.

**Figures 75–80.**
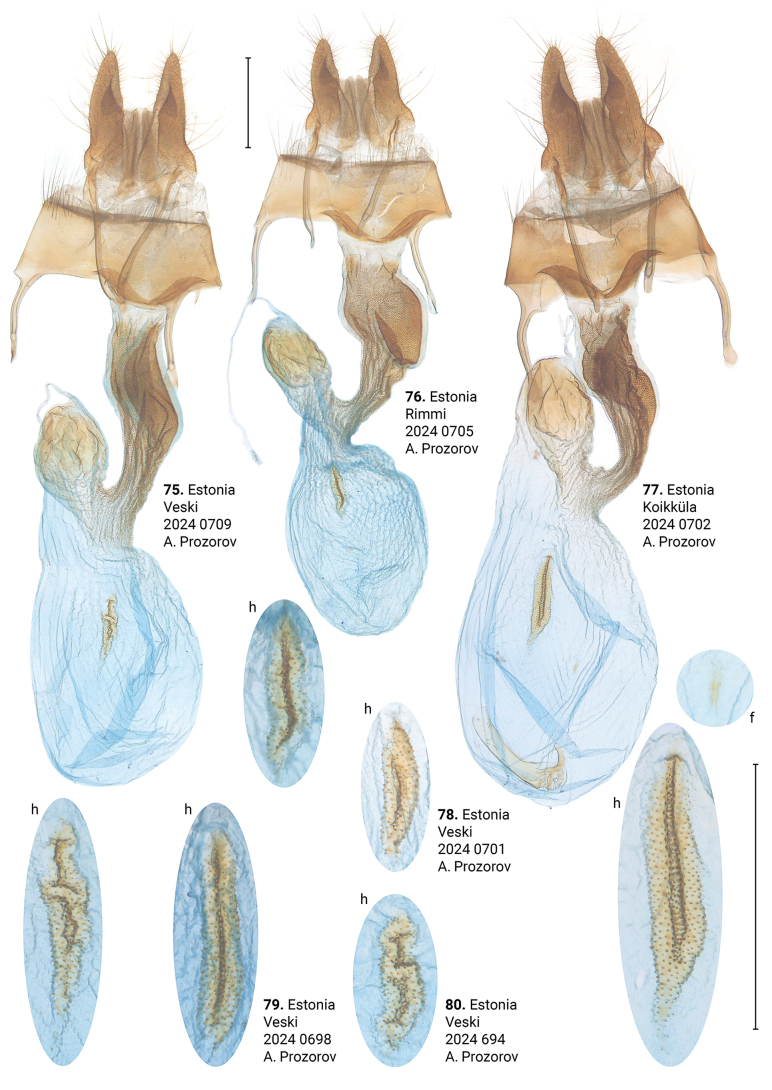
Female genitalia of *Xylomoiastrixstrix* (CRH). Abbreviations: f – frontal signum, h – hind signum. Scale bar: 1 mm.

**Figures 81–83.**
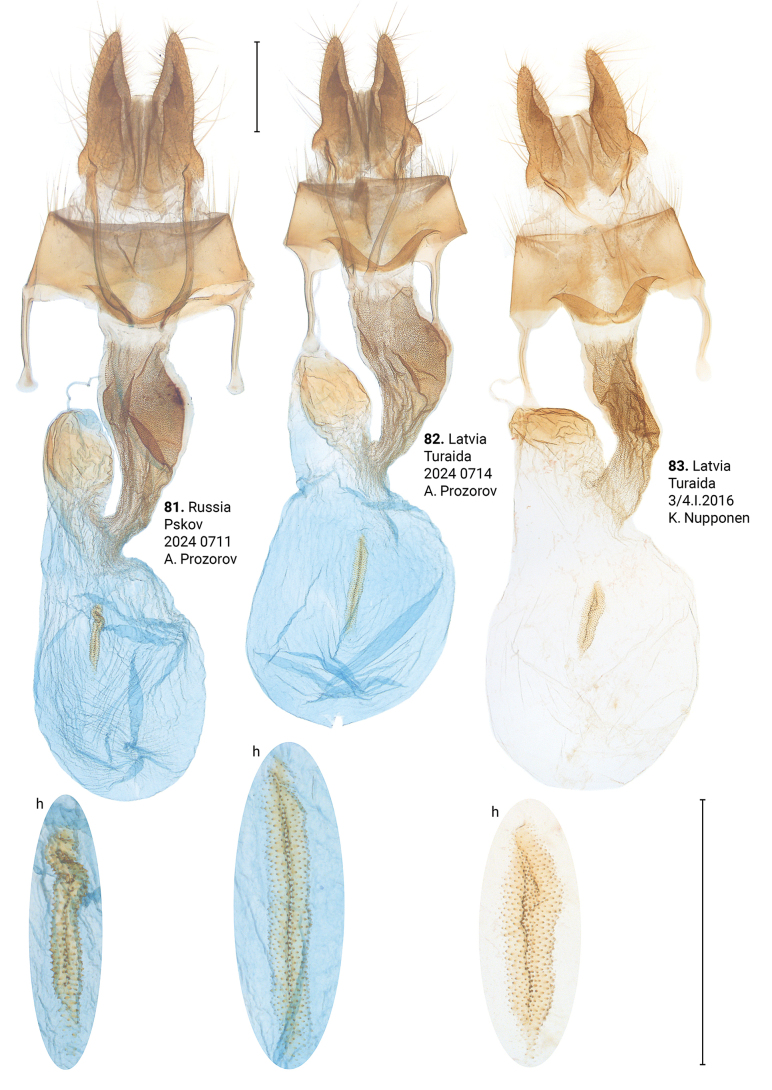
Female genitalia of *Xylomoiastrixstrix* (CRH). Abbreviations: f – frontal signum, h – hind signum. Scale bar: 1 mm.

**Figures 84–86.**
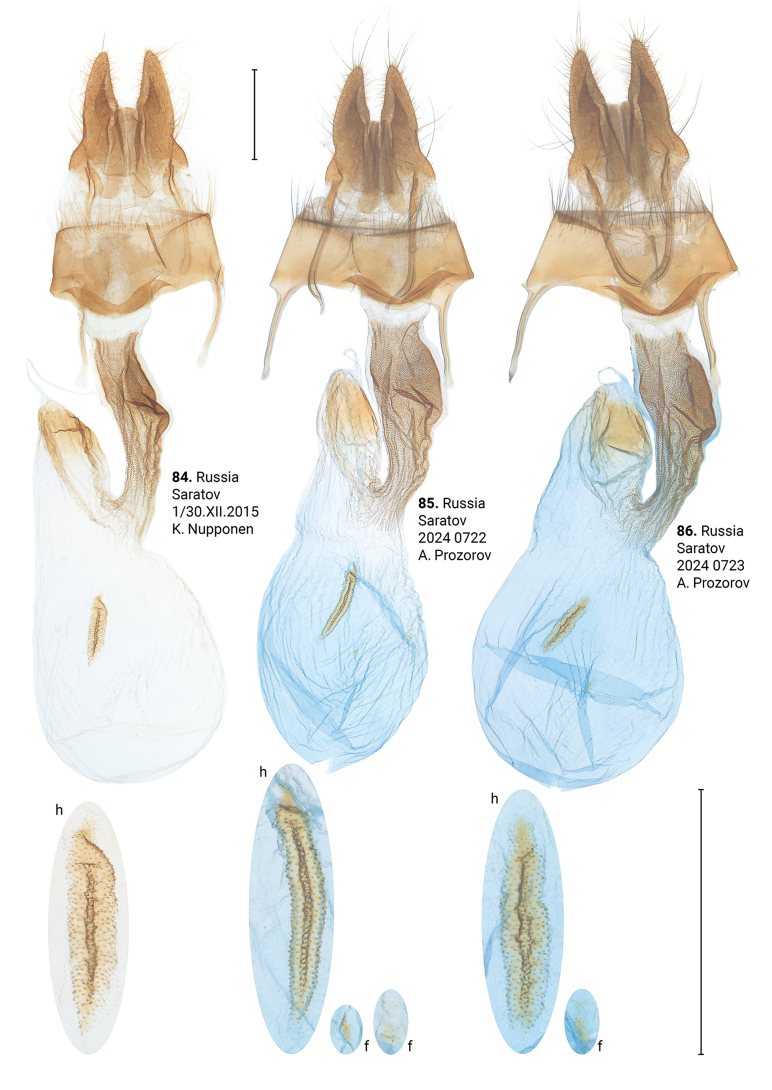
Female genitalia of *Xylomoiastrixstrix* (CRH). Abbreviations: f – frontal signum, h – hind signum. Scale bar: 1 mm.

**Figures 87–89.**
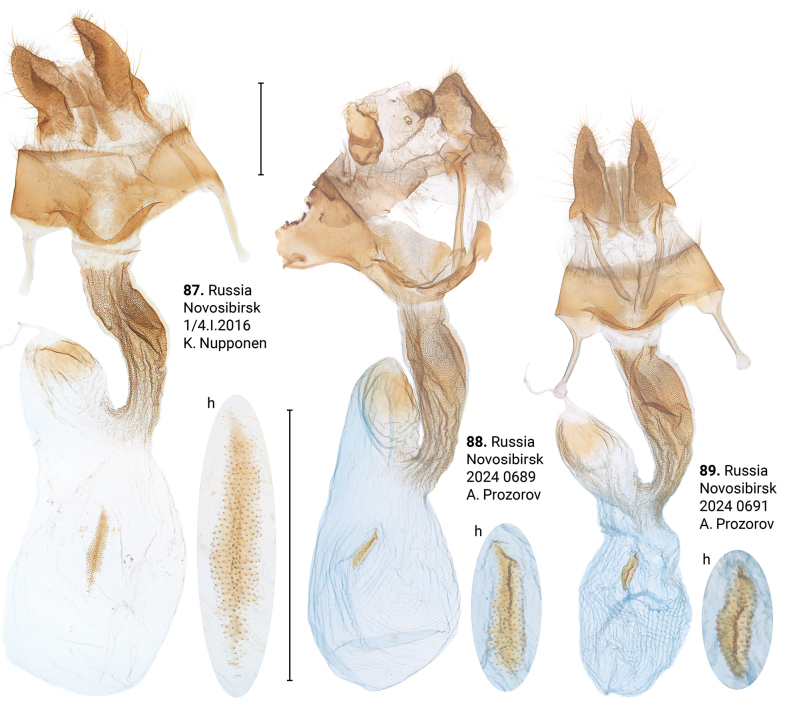
Female genitalia of *Xylomoiastrixretinax* (CRH). Abbreviations: h – hind signum. Scale bar: 1 mm.

#### Variability.

***Adults.*** May be dark-colored with brownish tinge (Figs 36–38) or pale-colored with yellowish tinge (Figs 39–41), submarginal area may be dark (e.g., Fig. 36) or pale (e.g., Fig. 38). ***Male genitalia.*** Uncus may gradually get thin towards apex (Figs 66, 68) or only be thin near its apex (Figs 65, 67), saccus varies in size, carina may be more (e.g., Fig. 65) or less pronounced (e.g., Fig. 67), additional cornutus similar to the basal one may be present near carina (Fig. 66), basal cornutus varies in size from small (e.g., Fig. 65) to large (e.g., Fig. 66), medial cornutus may be almost straight (Fig. 65) or c-shaped (e.g., Fig. 67). ***Female genitalia.*** Antevaginal plate slightly varies in thickness, bursa copulatrix and hind signum vary in size (Figs 87–89).

#### Distribution area.

Russia (Orenburg, Chelyabinsk, Omsk, Novosibirsk Oblasts and Altai Republic).

**Figure 90.**
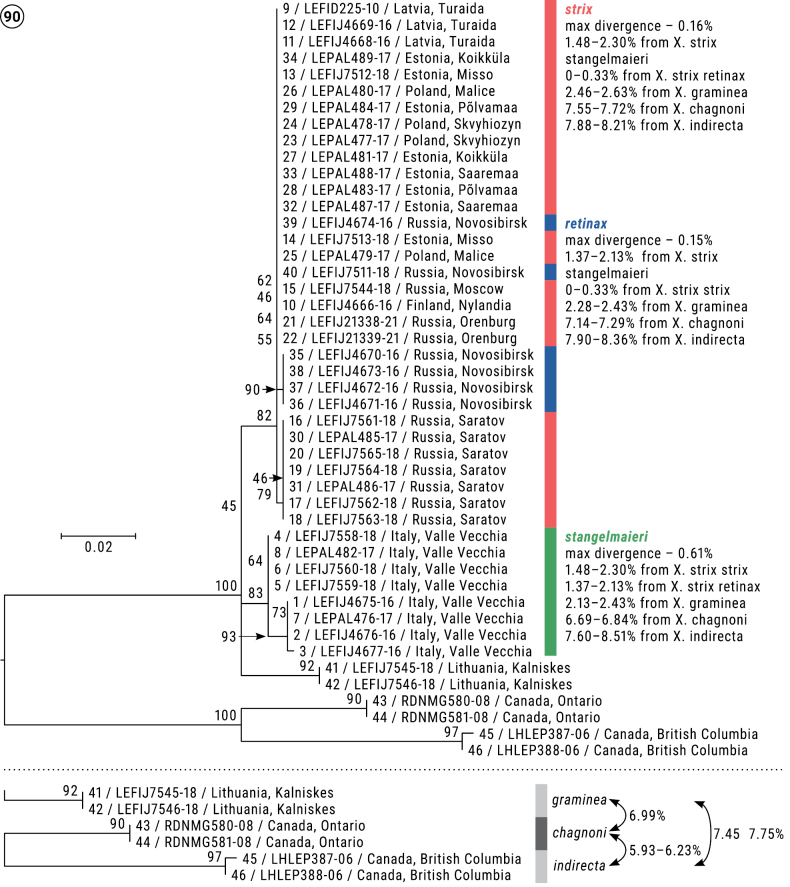
Phylogenetic tree (Maximum Likelihood, HKY+F+I, 1000 ultrafast bootstrap replicates) for *Xylomoia* spp. built in IQ-TREE 2.2.0 and pairwise distances (%) computed for each pair of taxa in MEGA X.

**Figures 91, 92.**
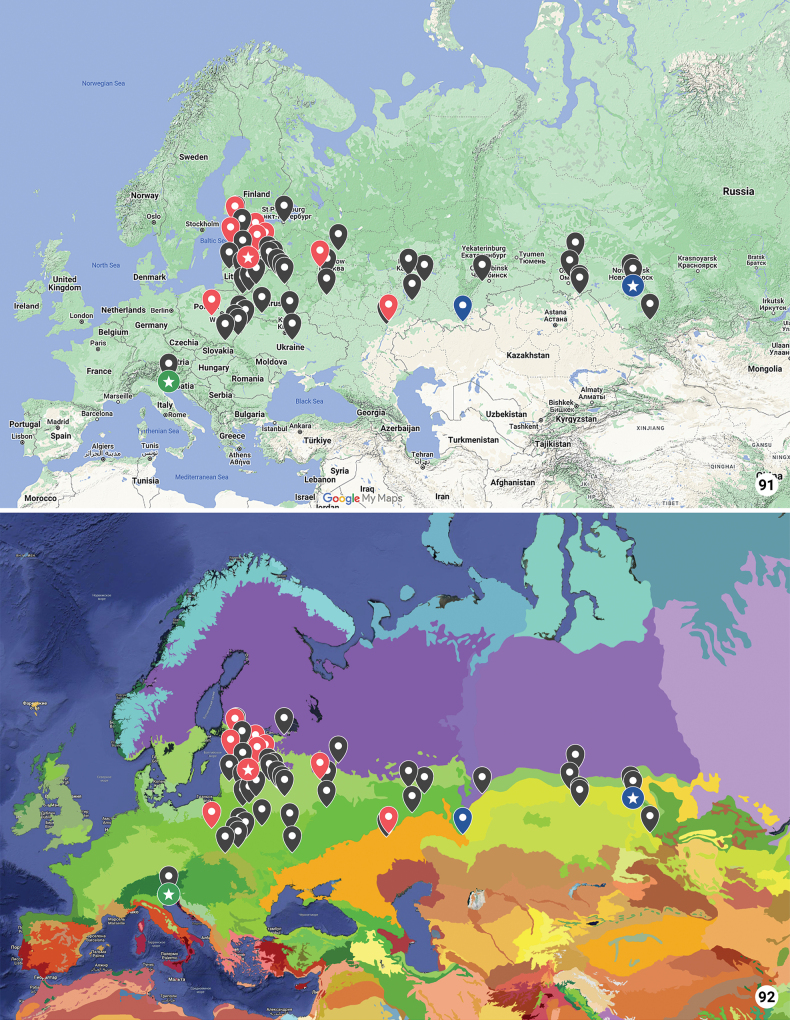
Collecting localities of *Xylomoiastrix* subspecies: *X.strixstangelmaieri* in Italy, *X.strixstrix* in Europe, and *X.strixretinax* in Asia. Colored tags mark collecting locations from where adults were barcoded. Circles with stars mark type localities **91** physical map **92** map of ecoregions: green colors indicate forests and steppes, purple – taiga (see ecoregions.appspot.com).

## Supplementary Material

XML Treatment for Xylomoia strix strix

XML Treatment for Xylomoia strix stangelmaieri

XML Treatment for Xylomoia strix retinax

## ﻿Additional information

### Author ORCIDs

Risto Haverinen https://orcid.org/0000-0001-8072-847X

Aleksander Pototski https://orcid.org/0000-0002-1843-3627

Marko Mutanen https://orcid.org/0000-0003-4464-6308

Darius Mikalauskas https://orcid.org/0009-0004-2145-8747

Roman V. Yakovlev https://orcid.org/0000-0001-9512-8709

Günter C. Müller https://orcid.org/0000-0002-7024-0179

Alexey M. Prozorov https://orcid.org/0000-0002-5668-0741

Aidas Saldaitis https://orcid.org/0000-0003-0999-3996

### Data availability

All of the data that support the findings of this study are available in the main text.

## References

[B1] Aarvik L Bengtsson BA Elven H Ivinskis P Jürivete U Karsholt O Mutanen M Savenkov N (2017) Nordic-Baltic Checklist of Lepidoptera. Norwegian Journal of Entomology Supplement 3: 1–236.

[B2] Ahola M Silvonen K (2007) Pohjoisen Euroopan yökkösten toukat [Larvae of Northern European Noctuidae]. Volume 1. Kuvaseppälä Yhtiöt Oy, Vaasa, 657 pp.

[B3] Anikin VV Sachkov SA Zolotuhin VV (2017) “Fauna Lepidopterologica Volgo-Uralensis”: from P. Pallas to present days. Proceedings of the Museum Witt Munich 7: 1–696.

[B4] Barnes W McDunnough J (1917) A new Canadian noctuid. Canadian Entomologist 49: 320–321. 10.4039/Ent49320-9

[B5] Bolshakov LV Makarichev NI (2020) Additions and corrections to the fauna of Lepidoptera of the Tula Province. 9. Eversmannia 61: 68–73.

[B6] Bury J Czudec P (2019) Comments on the occurrence and biology of *Xylomoiagraminea* (Graeser, 1889) (Lepidoptera: Noctuidae) from south-eastern Poland. Fragmenta Faunistica 62(1): 39–46. 10.3161/00159301FF2018.61.2.039

[B7] Bury J Zajda W (2012) Distribution of *Xylomoiagraminea* (Graeser, 1889) (Lepidoptera: Noctuidae) in Poland – review of previous studies and new data. Fragmenta Faunistica 55: 139–145. 10.3161/00159301FF2012.55.2.139

[B8] Buszko J (2004) Sówka puszczykówka. In: Adamski P Bartel R Bereszyski A Kapel A Witkowski Z (Eds) Gatunki Zwierząt (z wyjątkiem ptaków). Poradniki ochrony siedlisk i gatunków. Natura 2000. Podręcznik metodyczny.Ministerstwo Środowiska, Warszawa, 6: 63–64.

[B9] Buszko J (2010) Ksylomka striks (sówka puszczykówka) *Xylomoiastrix* Mikkola, 1980. In: Makomaska-Juchiewicz M (Ed.) Monitoring gatunków zwierząt. Przewodnik metodyczny. I. Biblioteka Monitoringu Środowiska. Inspekcja Ochrony Środowiska, Warszawa, 408 pp.

[B10] Cooksey C (2013) Quirks of dye nomenclature. 1. Evans blue. Biotechnic & Histochemistry, 89(2): 111–113. 10.3109/10520295.2013.822560 23957706

[B11] Council Directive (1992) Council Directive 92/43/EEC of 21 May 1992 on the conservation of natural habitats and of wild fauna and flora. Official Journal L 206, 22/07/1992, 0007–0050. http://data.europa.eu/eli/dir/1992/43/2013-07-01

[B12] Derzhinsky EA (2019) New findings of noctuid *Xylomoiastrix* (Lepidoptera, Noctuidae) in Belarus Poozerje. In: Science for education, production, economics. Materials of 71st regional scientific and practical conference of teachers, researchers and graduate students.Vitebsk 1: 42–43.

[B13] deWaard JR Ivanova NV Hajibabaei M Hebert PDN (2008) Assembling DNA Barcodes. In: Martin CC (Ed.) Environmental Genomics.Humana Press, Totowa, New Jersey, 275–294. 10.1007/978-1-59745-548-0_15 18642605

[B14] Dinerstein E Olson D Joshi A Vynne C Burgess ND Wikramanayake E Hahn N Palminteri S Hedao P Noss R Hansen M Locke H Ellis EC Jones B Barber CV Hayes R Kormos C Martin V Crist E Sechrest W Price L Baillie JEM Weeden D Suckling K Davis C Sizer N Moore R Thau D Birch T Potapov P Turubanova S Tyukavina A De Souza N Pintea L Brito JC Llewellyn OA Miller AG Patzelt A Ghazanfar SA Timberlake J Klöser H Shennan-Farpón Y Kindt R Barnekow Lillesø J-P Van Breugel P Graudal L Voge M Al-Shammari KF Saleem M (2017) An Ecoregion-Based Approach to Protecting Half the Terrestrial Realm. BioScience 1(6): 1–12. 10.1093/biosci/bix014 PMC5451287 28608869

[B15] Evans HM Schulemann W (1914) The action of vital stains belonging to the benzidine group. Science 39(1004): 443–454. 10.1126/science.39.1004.443 17808671

[B16] Geryak YuM Voytko PL Kanarsky YuV Chornyi TZ (2018) Supplement to the fauna of Noctuoidea (Lepidoptera, Insecta) of the Volyn Region. Scientific basis for preserving biotic diversity 9(16): 119–134.

[B17] Graeser L (1889) Beiträge zur Kenntniss der Lepidopteren-Fauna des Amurlandes. Berliner Entomologische Zeitung 33: 309–414. 10.1002/mmnd.47918880407

[B18] Grote AR (1875) Supplement to the list of North American Noctuidae. Bulletin of the Buffalo Society of natural History 2: 209–223.

[B19] Guenée A (1841) Noctuarum europaearum index methodicus, classificationis in Ann. Soc. entom gallic. editae tabulam fingens (1). Annales de la Société entomologique de France 10: 235–250.

[B20] Hacker H (1989) Beiträge zur systematischen Erfassung der Noctuidae des vorder- und zentralasiatischen Raumes. Neue taxonomische und faunistische Erkenntnisse zur Fauna Vorderasiens und Ägyptens (Lepidoptera, Noctuidae). Atalanta 19: 157–187.

[B21] Hardwick DF (1950) Preparation of slide mounts of lepidopterous genitalia. Canadian Entomologist 82(11): 231–235. 10.4039/Ent82231-11

[B22] Haverinen R Nupponen K Pototski A (2016) New data on the distribution and bionomics of *Xylomoiastrix* Mikkola, 1980 in the Baltic countries (Lepidoptera, Noctuidae). Lepinfo 22: 1–7.

[B23] Haverinen R Pototski A Matov A (2021) *Xylomoiastrix* (Lepidoptera, Noctuidae) recorded in Leningrad region, Russia. Lepinfo 24: 103–105.

[B24] Hebert PDN Cywinska A Ball SL deWaard JR (2003) Biological identifications through DNA barcodes. Proceedings of the Royal Society B 270(1512): 313–321. 10.1098/rspb.2002.2218 12614582 PMC1691236

[B25] Ivinskis P Rimšaitė J (2013) Data on new and rare Lepidoptera species for Lithuanian fauna. New and Rare for Lithuania Insect Species 25: 31–36.

[B26] Kalyaanamoorthy S Minh BQ Wong TKF Von Haeseler A Jermiin LS (2017) ModelFinder: Fast model selection for accurate phylogenetic estimates. Nature Methods 14: 587–589. 10.1038/nmeth.4285 28481363 PMC5453245

[B27] Karvonen J (1996) *Xylomoiastrix* jälleen Suomesta. Baptria 21: 51–52.

[B28] Klyuchko ZF Pljushch IG Sheshurak PN (2001) Annotated catalogue of noctuids (Lepidoptera, Noctuidae) of the Ukraine fauna. Zoology Institute of Ukrainian Academy of Sciences, Kiev, 884 pp.

[B29] Knyazev SA (2020) Catalogue of Lepidoptera of Omsk Oblast (Russia). Macrolepidoptera. Families: Hepialidae, Brachodidae, Cossidae, Sesiidae, Limacodidae, Zygaenidae, Thyrididae, Drepanidae,Uraniidae, Geometridae, Lasiocampidae, Lemoniidae, Endromididae, Saturniidae, Sphingidae,Notodontidae, Lymantriidae, Arctiidae, Syntomidae, Erebidae, Nolidae, Noctuidae, Hesperiidae, Papilionidae, Pieridae, Lycaenidae, Nymphalidae, Satyridae. Acta Biologica Sibirica 6: 139–226. 10.3897/abs.6.e53005

[B30] Knyazev SA Ivonin VV Dubatolov VV Vasilenko SV Ponomaryov KB (2015) New records of Lepidoptera from the South of West Siberia. Amurian zoological journal VII(1): 43–50. 10.33910/1999-4079-2015-7-1-43-50

[B31] Knyazev SA Ivonin VV Vasilenko SV (2016) New and interesting findings of butterflies and moths (Insecta, Lepidoptera) in Omsk and Novosibirsk provinces. Amurian zoological journal VIII (4): 254–272. 10.33910/1999-4079-2016-8-4-254-272

[B32] Kononenko VS (2016a) Noctuidae. In: Leley AS (Ed.) Annotated catalogue of the insects of Russian Far East. Volume II. Lepidoptera. Vladivostok, Dalnauka, 812 pp.

[B33] Kononenko VS (2016b) Noctuidae: Cuculliinae – Noctuinae, part (Lepidoptera). Noctuoidea Sibiricae. Part 3. Proceedings of the Museum Witt Munich 5: 1–497.

[B34] Kumar S Stecher G Li M Knyaz Ch Tamura K (2018) MEGA X: Molecular Evolutionary Genetics Analysis across Computing Platforms. Molecular Biology and Evolution 35(6): 1547–1549. 10.1093/molbev/msy096 29722887 PMC5967553

[B35] Lafontaine JD Schmidt BCh (2010) Annotated check list of the Noctuoidea (Insecta, Lepidoptera) of North America north of Mexico. ZooKeys 40: 1–239. 10.3897/zookeys.40.414 PMC3234417 22207802

[B36] Lederer J (1857) Die Noctuinen Europa’s mit Zuziehung einiger bisher meist dazu gezählter Arten des asiatischen Russland’s, Kleinasien’s, Syrien’s und Labrador’s. Friedrich Manz, Wien, 251 pp. 10.5962/bhl.title.60460

[B37] Matov AYu Kononenko VS Sviridov AV (2019) In: Sinev SYu (Ed.) Catalogue of the Lepidoptera of Russia. Second edition. Zoological Institute RAS, St. Petersburg, 448 pp.

[B38] Matov AYu Kononenko VS Sviridov AV (2023) Catalogue of the Lepidoptera of Russia [online version] Ver. 2.3. https://www.zin.ru/publications/books/Lepidoptera_Russia

[B39] Mikkola K (1980) Two new noctuid species from Northern Europe: *Poliasabmeana* n. sp. and *Xylomoiastrix* n. sp. (Lepidoptera, Noctuidae: Hadeninae and Amphipyrinae). Notulae Entomologicae 60: 217–222.

[B40] Mikkola K (1998) Revision of the genus *Xylomoia* Staudinger (Lepidoptera: Noctuidae), with descriptions of two new species. Systematic Entomology 23: 173–186. 10.1046/j.1365-3113.1998.00055.x

[B41] Minh BQ Schmidt HA Chernomor O Schrempf D Woodhams MD Von Haeseler A Lanfear R Teeling E (2020) IQ-TREE 2: New Models and Efficient Methods for Phylogenetic Inference in the Genomic Era. Molecular Biology and Evolution 37: 1530–1534. 10.1093/molbev/msaa015 32011700 PMC7182206

[B42] Nowacki J Pałka K (2014) Nowe stanowisko *Xylomoiastrix* Mikkola, 1980 (Lepidoptera: Noctuidae) w Polsce. Wiadomości entomologiczne 33: 38–41.

[B43] Nowacki J Sekuła W (1994) *Xylomoiastrix* Mikkola, 1980 – nowy dla fauny Polski przedstawiciel sówkowatych (Lepidoptera, Noctuidae). Wiadomości Entomologiczne 13(3): 195–196.

[B44] Nupponen K Fibiger M (2002) Contribution to the knowledge of the fauna of Bombyces, Sphinges and Noctuidae of the Southern Ural Mountains, with description of a new *Dichagyris* (Lepidoptera: Lasiocampidae, Endromidae, Saturniidae, Sphingidae, Notodontidae, Noctuidae, Pantheidae, Lymantriidae, Nolidae, Arctiidae). Phegea 30(4): 121–185.

[B45] Pekarsky ON Korb SK (2012) The first finding of *Xylomoiaretinax* Mikkola, 1998 in Lover Volga region (Lepidoptera: Noctuidae). Eversmannia 31–32: 114.

[B46] Pierce FN (1909) The genitalia of the group Noctuidae of the Lepidoptera of the British Islands: An account of the morphology of the male clasping organs. A.W. Duncan, Liverpool, 88 pp. 10.5962/bhl.title.8998

[B47] Ratnasingham S Hebert PD (2007) BOLD: The Barcode of Life Data System (http://www.barcodinglife.org). Molecular Ecology Notes 7(3): 355–364. 10.1111/j.1471-8286.2007.01678.x PMC1890991 18784790

[B48] Ratnasingham S Hebert PD (2013) A DNA-Based Registry for All Animal Species: The Barcode Index Number (BIN) System. PLoS ONE 8(8): e66213. 10.1371/journal.pone.0066213 PMC3704603 23861743

[B49] Rockburne EW Lafontain JD (1976) The cutworm moths of Ontario and Quebec. Printing and Publishing Supply and Services Canada, Ottawa, 164 pp.

[B50] Sachkov SA (2013) The new for Samara area species of Lepidoptera. 4^th^ report. Bulletin of Samara University 3(104): 188–199. 10.18287/2541-7525-2013-19-3-188-199

[B51] Savenkov N Šulcs I (2010) Latvijas Tauriņi. Katalogs. Tallinn, 176 pp.

[B52] Skou P (1991) Nordens ugler. Danmarks dyreliv. Bind 5. Stensrup, 566 pp.

[B53] Sugi S (1976) A new species of the genus *Xylomoia* Staudinger (Lepidoptera, Noctuidae, Amphipyrinae). Tinea 10: 63–66.

[B54] Šulcs A Šulcs I (1983) Zur Kenntnis von *Xylomoiastrix* Mikkola, 1980 (Lep., Noctuidae). Entomologische Nachrichten und Berichte 27(5): 227–228.

[B55] Sviridov AV (2002) Noctuid moths (Lepidoptera) new for different areas of the Russian Federation, 1. Russian Entomological Journal 11(4): 445–450.

[B56] Ūsaitis T Mikalauskas D Bačianskas V (2019) New and rare for the Lithuanian fauna Lepidoptera species recorded in 2019. Bulletin of the Lithuanian Entomological Society 3(31): 79–98.

[B57] Volynkin AV (2024) On the terminology of the genitalia structures of lichen moths (Lepidoptera: Erebidae: Arctiinae: Lithosiini) with some references to Noctuidae. Ecologica Montenegrina 73: 176–207. 10.37828/em.2024.73.18

[B58] Volynkin AV Ivanova MS (2016) First record of *Xylomoiaretinax* Mikkola, 1998 (Lepidoptera: Noctuidae) from Altai Republic, Russia. Far Eastern Entomologist 324: 13–14.

[B59] Zilli A Ronkay L Fibiger M (2005) Noctuidae Europeae. Volume 8. Apameini. Entomological Press, Sorø, 323 pp.

